# The Urate-Lowering Effects and Renal Protective Activity of Iridoid Glycosides from *Paederia foetida* in Rats with Hyperuricemia-Induced Kidney Injury: A Pharmacological and Molecular Docking Study

**DOI:** 10.3390/molecules30153098

**Published:** 2025-07-24

**Authors:** Haifeng Zhou, Xinyi Yue, Longhai Shen, Lifeng Wu, Xiaobo Li, Tong Wu

**Affiliations:** 1 School of Pharmacy, Shanghai Jiao Tong University, Shanghai 200240, China; xfphoenix@163.com (H.Z.); xbli@sjtu.edu.cn (X.L.); 2 National Key Laboratory of Lead Druggability Research, Shanghai Institute of Pharmaceutical Industry, China State Institute of Pharmaceutical Industry, Shanghai 201203, China; eyeeye88@163.com (X.Y.); shenlh28@163.com (L.S.); 3 Ningo Dachang Pharmaceutical Co., Ltd., Ningbo 201203, China; foxwlf@hotmail.com

**Keywords:** iridoid glycosides, *Paederia foetida* Linn., hyperuricemia, uric acid transporters, renoprotective effect, molecular docking

## Abstract

(1) Background: The urate-lowering effects of three iridoid glycosides, which are paederosidic acid, paederosidic acid methyl ester, and paederoside, isolated from *Paederia foetida* and the protection they provide against hyperuricemia-induced kidney injury were investigated in a rat model. (2) Methods: A hyperuricemia (HUA) rat model was established in Sprague-Dawley (SD) rats through intraperitoneal potassium oxonate (PO) and intragastrical adenine for 2 weeks. Subsequently, rats in the pharmaceutical intervention groups received corresponding drug treatments at a concentration of 40 mg/kg/day, maintained consistently for 7 days. (3) Results: The results showed that three compounds reduced serum urate (SU), creatinine (CRE), and blood urea nitrogen (BUN) levels and that the urinary excretion levels of uric acid, urine urea nitrogen, and creatinine increased. Furthermore, the administration of three iridoid glycosides enhanced renal filtration capacity, as demonstrated by the elevated 24 h creatinine clearance rate (CCR) and 24 h uric acid clearance rate (CUA); improved the fraction excretion of uric acid (FEUA); and attenuated renal damage. Finally, three iridoid glycosides promoted uric acid excretion in HUA rats by downregulating URAT1 and GLUT9 and upregulating ABCG2, OAT1, and OAT3. Moreover, the molecular docking results further corroborated the finding that the three compounds can bind to multiple sites of the uric acid transporter via hydrogen, P-π, and hydrophobic bonds. (4) Conclusions: The three iridoid glycosides were found to lower SU levels by increasing uric acid excretion. They are promising natural products for the prevention of HUA and HUA-induced kidney injury.

## 1. Introduction

Uric acid (UA) is the final product of purine metabolism in humans [1]. Its concentration within the range of 180 to 420 μM (3.0 to 7.0 mg/dL) is generally considered normal [2]. In cases of hyperuricemia, a blood uric acid level exceeding 420 μM (7.0 mg/dL), especially when reaching or exceeding 700 μM (approximately 12 mg/dL), is associated with an increased risk of gout or acute kidney injury [3].

Hyperuricemia is a metabolic disorder typically characterized by elevated blood uric acid levels. In recent years, accumulating evidence has indicated that impaired uric acid excretion is often associated with hyperuricemia [4]. The U.S. National Health and Nutrition Examination Survey (NHANES) indicated that approximately 3.9% of adults have been diagnosed with hyperuricemia [5]. According to a survey covering 13 provinces in China, the prevalence of hyperuricemia among adults was found to be 6.4% [6]. Hyperuricemia could induce kidney dysfunction and contribute to kidney disease progression through a number of potential mechanisms [7]. Thus, controlling hyperuricemia is an important strategy to reduce kidney disease risk [8].

Clinical studies have shown that impaired uric acid excretion is the main cause of hyperuricemia in gout patients, which is often associated with kidney dysfunction [9]. The kidneys’ handling of uric acid encompasses glomerular filtration, tubular reabsorption, tubular secretion, and post-secretory reabsorption [10]. A decline in renal function impairs uric acid excretion, which may lead to elevated serum urate levels and the onset of hyperuricemia. Renal uric acid excretion disorders constitute the primary etiology of persistent hyperuricemia in approximately 90% of affected individuals. When uric acid levels exceed the saturation threshold, uric acid and precipitated urate crystals may deposit within the renal tubules and interstitium, resulting in renal injury [11]. The kidneys serve as the primary site for the excretion and reabsorption of uric acid. Uric acid transporters, including URAT1 (SLC22A12) [12] and GLUT9 (SLC2A9) [13], facilitate the glomerular free filtration of uric acid. The most important secretory transporter is ABCG2 [14]. Additionally, OAT1 (SLC22A6) and OAT3 (SLC22A8) [15] function as the major proteins responsible for urate salt secretion, while GLUT9 also plays a critical role in uric acid reabsorption.

*Paederia foetida* Linn. (syn. *Paederia scandens* (Lour.) Merrill) is a climbing plant of the Rubiaceae family and Paederia genus. It is also identified as skunk vine, stink vine, or Chinese fever vine. It is popularly known as “Ji Shi Teng” in China [16]. It is widely grown in India [17,18], Japan, Korea [19], the Philippines, Vietnam [20], and the USA [20,21]. It has been traditionally used as a medicinal plant and an edible plant in Southeast Asia for thousands of years [22,23]. For instance, people in Hainan province of China make paederia snacks such as paederia dumplings, paederia rice noodles, and paederia cakes [24,25,26]. The plant is abundantly consumed in Northeast India and used in Thai cuisine [27].

In recent years, an increasing number of studies have focused on the role of the extracts of *Paederia foetida* Linn. in lowering uric acid levels and protecting the kidneys [28,29,30,31]. In our previous studies, we extracted total iridoid glycosides from *Paederia scandens* (Lour.) Merrill. comprising approximately 32% paederosidic acid (JST-1), 11% paederosidic acid methyl ester (JST-2), and 6% paederoside (JST-3). The results indicated that the administration of total iridoid glycosides exhibited notable therapeutic efficacy in ameliorating adenine-induced uric acid nephropathy in rats [32]. JST-1 and JST-2 were recognized for their various pharmacological activities, including their ability to inhibit tumor growth [33] and suppress inflammation and the fact that they have anti-microbial properties [34], antinociceptive activity [35], and anticonvulsant and sedative effects [36]. To the best of our knowledge, to date, research has only reported the uric acid-lowering activity of the extract of *Paederia foetida* Linn. [28,29,30,31], whereas the uric acid-lowering potential of its constituent monomer compounds has not yet been investigated or documented.

In the present study, the combination of potassium oxonate and adenine was used to induce hyperuricemia in rats. This animal model was used to investigate the uric acid-lowering and renoprotective effects and the possible mechanisms of three iridoid glycosides obtained from *Paederia foetida* Linn. The goal is to offer a natural source for the prevention and treatment of HUA.

## 2. Results

### 2.1. The Effects of Three Iridoid Glycosides on Acute Hyperuricemia

**Figure 1:**
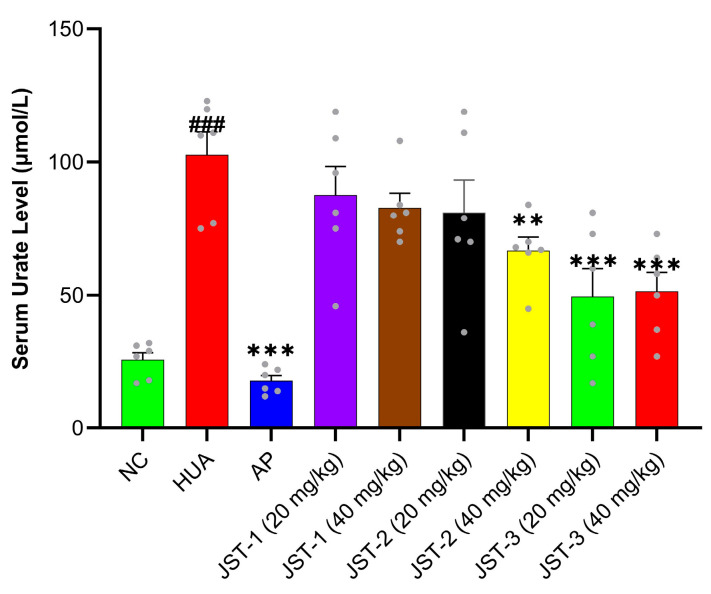
Effects of three iridoid glycosides on serum urate level in acute hyperuricemia induced by potassium oxonate. Data are presented as mean ± SEM, ### *p* < 0.001 vs. NC group; ** *p* < 0.01, *** *p* < 0.001 vs. HUA group.

As shown in Figure 1, the serum urate level increased from 25 μM in the NC group to 100 μM in the HUA group, indicating the successfulness of the model’s development. Compared to the HUA group, treatment with JST-2 at 40 mg/kg significantly reduced the serum urate levels (*p* < 0.01). JST-3 at both low and high concentrations exhibited even greater urate-lowing effects (*p* < 0.001). Allopurinol (AP), as the positive control, demonstrated a significant inhibitory effect on acute hyperuricemia (*p* < 0.001). The low and high doses of JST-1, as well as the low dose of JST-2, showed a trend toward reducing urate levels but was not statistically significant.

### 2.2. The Effects of Three Iridoid Glycosides on Body Weight, Kidney Weight, and Organ Coefficients in HUA Rats

The body weight of rats was measured on the 7th, 10th, 14th, and 21st days; however, in the initial phase of the experiment (the first 10 days), no statistically significant differences in body weight were detected among the groups. From the 14th day onward, the body weights of the rats in the HUA group were significantly lower (*p* < 0.05) than those in the NC group, whereas at day 21, after one week of continuous administration, the body weights were significantly elevated (*p* < 0.001) in both the groups treated with the three iridoid glycosides and allopurinol-treated groups, and they were also significantly elevated (*p* < 0.01) in the benzbromarone (BM) group compared to the HUA group.

**Figure 2:**
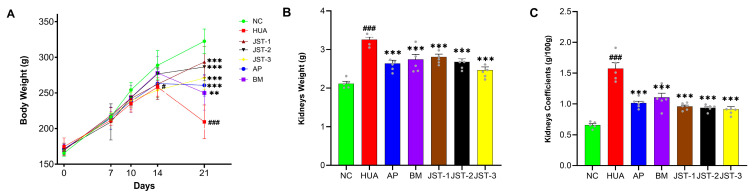
Effects of three iridoid glycosides on body weight, kidney weight, and organ coefficients in HUA rats. (**A**) Body weight was measured on 7th, 10th, 14th, and 21st days. (**B**) Kidney weight. (**C**) Organ coefficients for kidneys. Data are presented as mean ± SEM, # *p* < 0.05, ### *p* < 0.001 vs. NC group; ** *p* < 0.01, *** *p* < 0.001 vs. HUA group.

At the end of the experiment, the weight of the kidneys in HUA rats increased by approximately 1.54-fold compared to that in the NC group, whereas about 1.25-, 1.30-, 1.33-, 1.27-, and 1.16-fold decreases were observed after treatments with AP, BM, JST-1, JST-2, and JST-3, respectively (Figure 2B). In addition, there were significant alterations in terms of the kidney coefficient (Figure 2C) among the HUA and administration groups. The increase in the kidney index induced by high uric acid levels was reversed after the intervention of the administration groups (*p* < 0.01). In particular, the renal coefficient of the groups treated with the three iridoid glycosides decreased by approximately 30% of that observed in the HUA group, which demonstrated a slightly superior effect compared to AP and BM.

It was found that the kidneys of the HUA group were significantly enlarged, and the color of the kidneys changed from dark red to dark brown visible to the naked eye. Some kidneys showed milky white granular crystals. After drug treatment, the renal lesions of rats in each administration group demonstrated significant amelioration compared to those in the HUA group.

### 2.3. The Effects of Three Iridoid Glycosides on Renal Function Indicators in HUA Rats After Single-Dose Administration

**Figure 3:**
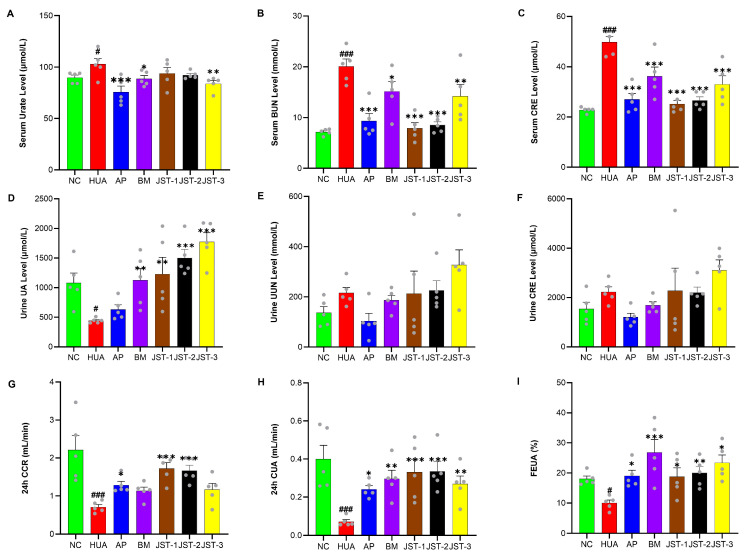
The effects of three iridoid glycosides on renal function indicators in HUA rats after single-dose administration. (**A**) Serum urate level; (**B**) serum blood urea nitrogen (BUN) level; (**C**) serum creatinine (CRE) level; (**D**) urine uric acid (UA) level; (**E**) urine urea nitrogen (UUN) level; (**F**) urine creatinine (CRE) level; (**G**) 24 h creatinine clearance rate (CCR); (**H**) 24 h uric acid clearance rate (CUA); (**I**) fraction excretion of uric acid (FEUA %). Data are presented as the mean ± SEM, # *p* < 0.05, ### *p* < 0.001 vs. NC group; * *p* < 0.05, ** *p* < 0.01, *** *p* < 0.001 vs. HUA group.

The SU, BUN, and CRE contents were determined to assess the protective effects of three iridoid glycosides on renal injury in HUA rats. After single-dose administration, the levels of SU, BUN, and CRE were obviously increased in the HUA group in comparison to the NC group (*p* < 0.05, *p* < 0.001, *p* < 0.001) (Figure 3A–C). These results indicated that the HUA model was successfully constructed, and the kidneys had been damaged.

Compared to the HUA group, all three iridoid glycosides significantly reduced serum CRE levels (*p* < 0.001). Among them, JST-1 and JST-2 were slightly more enhanced than AP in terms of reducing serum CRE and significantly superior to JST-3 and BM. The trend in the effects of these three compounds on serum BUN was similar to that for serum CRE. The 24 h creatinine clearance rate (24 h CCR) in the HUA group was significantly reduced, which reduced to 30% in the NC group, indicating severe kidney damage. However, JST-1 and JST-2 significantly (*p* < 0.001) increased the 24 h CCR, which was about 2.5 times that of the HUA group; meanwhile AP only significantly (*p* < 0.05) increased the 24 h CCR, which was about 1.8 times that of the HUA group. There was no significant difference in the 24 h CCR among JST-3 and BM. The 24 h uric acid clearance rate (24 h CUA) in the HUA group was significantly reduced, which was decreased to 1/5 of the NC group. JST-1 and JST-2 significantly (*p* < 0.001) increased the 24 h CUA to approximately 80% of the NC group. JST-3 and BM significantly (*p* < 0.01) increased the 24 h CUA, which increased by 65% and 75%, respectively, while AP only significantly (*p* < 0.05) increased the 24 h CUA. All treatment groups could significantly increase the uric acid excretion fraction percent (FEUA%). The highest FEUA was 26.8% in the BM group, followed by that in the JST-2 group.

### 2.4. The Effects of Three Iridoid Glycosides on Renal Function Indicators in HUA Rats After One Week of Administration

**Figure 4:**
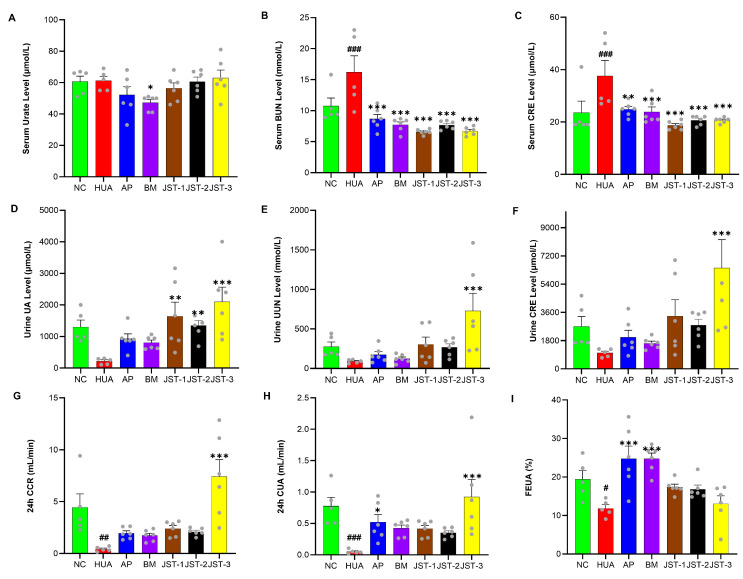
The effects of three iridoid glycosides on renal function indicators in HUA rats after one week of administration. (**A**) Serum urate level; (**B**) serum blood urea nitrogen (BUN) level; (**C**) serum creatinine (CRE) level; (**D**) urine uric acid (UA) level; (**E**) urine urea nitrogen (UUN) level; (**F**) urine creatinine (CRE) level; (**G**) 24 h creatinine clearance rate (CCR); (**H**) 24 h uric acid clearance rate (CUA); (**I**) fraction excretion of uric acid (FEUA %). Data are presented as the mean ± SEM, # *p* < 0.05, ## *p* < 0.01, ### *p* < 0.001 vs. NC group; * *p* < 0.05, ** *p* < 0.01, *** *p* < 0.001 vs. HUA group.

**Figure 5:**
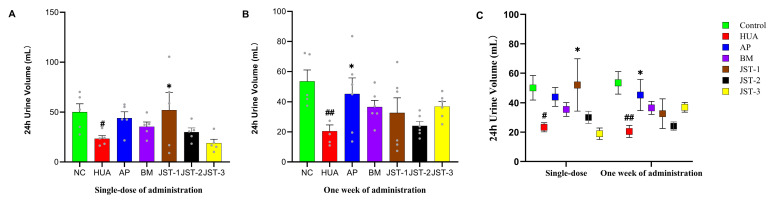
Urine output on day 1 and day 7 after drug administration. (**A**) The 24 h urine volume after single-dose administration; (**B**) 24 h urine volume after one week of administration; (**C**) A and B correlation chart. Data are presented as the mean ± SEM, # *p* < 0.05, ## *p* < 0.01 vs. NC group; * *p* < 0.05 vs. HUA group.

After one week of administration, the serum urate levels in all treatment group returned to baseline levels comparable to those of the NC group (Figure 4A). However, urinary uric acid levels remained significantly higher than those of the NC group (Figure 4D). Based on the analysis of urine output, it was observed that the 24 h urine volume in the three iridoid glycosides groups was lower than that of the NC group (Figure 5).

After one week of continuous administration, renal function indicators in the serum and urine of HUA rats were tested to evaluate the protective effects of three iridoid glycosides on kidney damage caused by hyperuricemia. As shown in Figure 4, after one week of continuous administration, the levels of serum BUN and serum CRE in the HUA group rats were significantly higher than those of the NC group (*p* < 0.001), while the levels of these indicators were significantly reduced in each treatment group (*p* < 0.001), which was basically equivalent to the NC group. The efficacy of the three iridoid glycosides was marginally superior to that observed in the two positive control groups. In terms of blood uric acid content, a significant difference was only found between the BM group and the HUA group (*p* < 0.05). The results of renal indicators in urine showed that the urine uric acid content in the JST-3 group was significantly higher than that in the HUA group (*p* < 0.001), and the urine uric acid level in the JST-1 and JST-2 groups was also significantly higher than that in the HUA group (*p* < 0.01 and *p* < 0.05). The urine urea nitrogen and creatinine levels in the JST-3 group were also significantly (*p* < 0.001) higher than those in the HUA group, while other treatment groups did not show significant changes in these two renal indicators compared to the HUA group. As for the two indicators of the 24 h CCR and 24 h CUA, those of the JST-3 group were significantly (*p* < 0.001) higher than those of the HUA group, and those of all other treatment groups were higher than those of the HUA group, but there was no significant difference. The positive control groups of AP and BM could significantly increase the FEUA to about 24.8%, followed by the JST-1 group with 17.4%, the JST-2 group with 16.8%, and the JST-3 group with 13.0%; however, there were no significant differences compared to the HUA group, for which the FEUA was 11.9%.

### 2.5. Pathological Observation of Kidney Tissues

**Figure 6:**
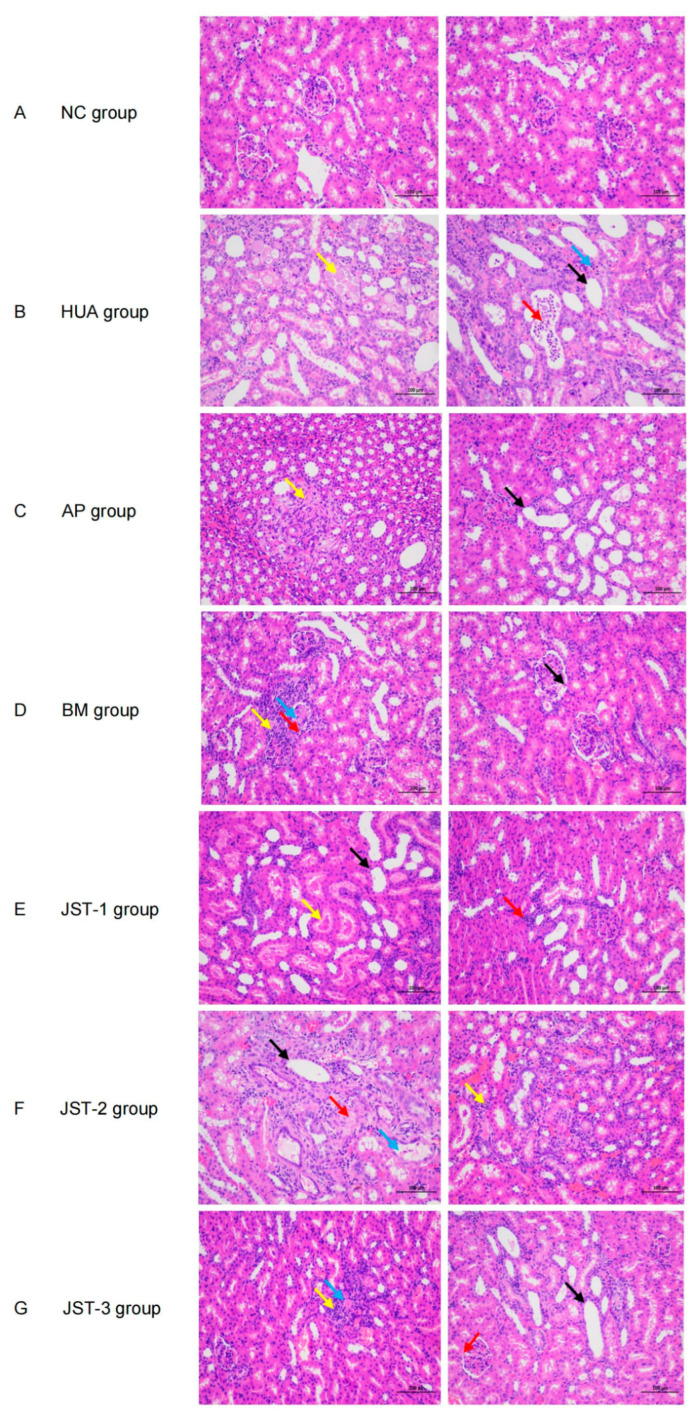
The effects of three iridoid glycosides on renal histopathological alterations in HUA-induced renal injury rats. (**A**) The normal (NC) group; (**B**) the hyperuricemia (HUA) group; (**C**) the allopurinol (AP) group; (**D**) the benzbromarone (BM) group; (**E**) the JST-1 group; (**F**) the JST-2 group; (**G**) the JST-3 group. Black arrows: tubular dilation; yellow arrows: urate deposits; red arrows: inflammatory cell infiltrations; blue arrows: lymphocyte infiltration.

H&E staining revealed histopathological damage in the rats with hyperuricemia. In the normal control group, the boundary between the renal cortex and medulla of the rats was clear, the morphology and structure of the glomeruli were normal, the renal tubular epithelial cells were tightly arranged, and no atrophy or vacuolation was observed. No protein or urate crystal deposition was observed in the tubules, and no inflammatory cell infiltration was detected in the interstitium. However, in the HUA group, significant tubular dilation was observed in both the renal cortex and medulla. Localized tubular lumens contained abundant urate deposits, along with a small number of inflammatory cell and lymphocyte infiltrations (Figure 6B), which shows significant pathological differences compared to the normal control group, indicating successful modeling. These pathological changes were improved after treatment with three iridoid glycosides. It was observed that the deposition of urate salts and glomerular lesions in the kidneys were reduced, and the degree of tubular dilation was also improved compared to the HUA group. Notably, JST-1 and JST-2 performed exceptionally well in alleviating urate deposition, with effects comparable to those of the positive control group (Figure 6E,F).

### 2.6. The Effects of Three Iridoid Glycosides on Urate Transporter Protein Expression in the Kidneys

**Figure 7:**
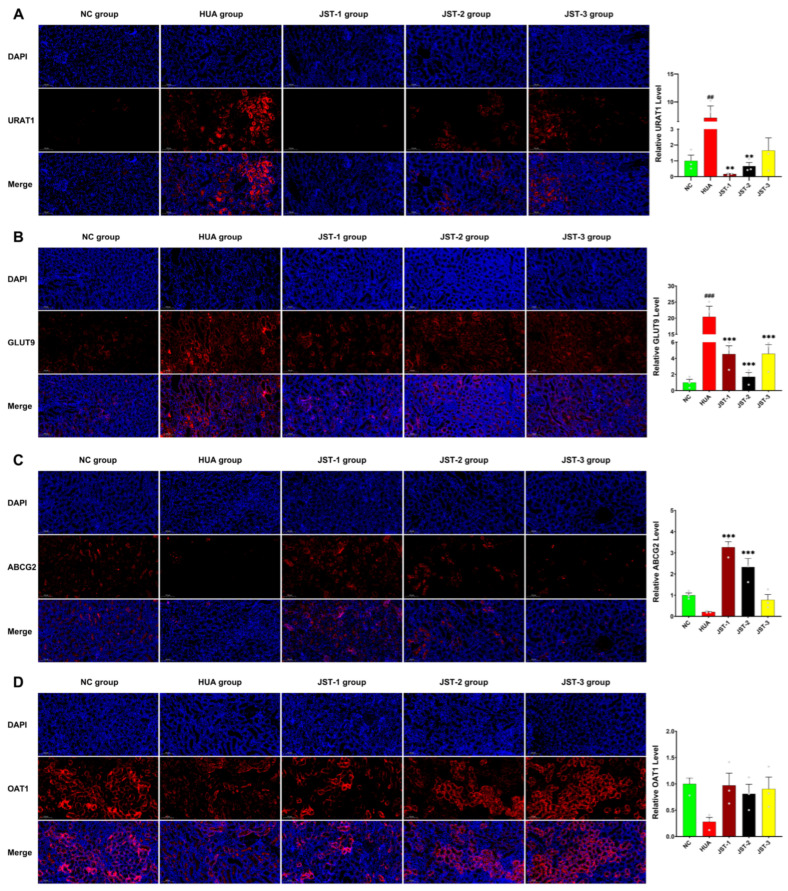

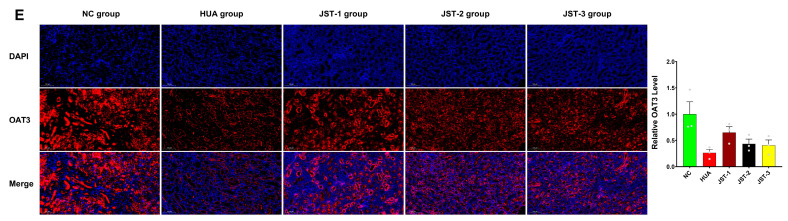
Immunofluorescence staining visualized the expression of key urate transporters in renal tissue: URAT1 (**A**), GLUT9 (**B**), ABCG2 (**C**), OAT1 (**D**), and OAT3 (**E**) (scale bar = 100 μm). The relative positive area ratio for each transporter was quantified to assess expression levels. All transporters exhibited distinct membrane-localized signals (red), with nuclei counterstained by DAPI (blue). Data are presented as the mean ± SEM, ## *p* < 0.01, ### *p* < 0.001 vs. NC group; ** *p* < 0.01, *** *p* < 0.001 vs. HUA group.

Renal urate transporters play a principal role in uric acid excretion. Compared with the NC group, the proteins of reabsorption transporters URAT1 and GLUT9 were upregulated, whereas the secretory transporter proteins ABCG2, OAT1, and OAT3 were downregulated in the renal tissue of the HUA group (Figure 7). Immunofluorescence analysis further verified the role of JST-1, -2, and -3 in regulating urate transporters. As shown in Figure 7A, the analysis of the positive area ratio of URAT1 protein expression demonstrated significantly higher levels in the HUA group than in the NC group. In comparison to the HUA group, both the JST-1 and JST-2 groups exhibited a significant reduction in the relative positive area ratio of URAT1 expression (*p* < 0.01), with protein levels even lower than those in the NC group. As shown in Figure 7B, all three treatment groups showed significantly reduced GLUT9 protein expression levels compared to the HUA group, though they remained higher than those in the NC group. As depicted in Figure 7C, the fluorescence intensity of ABCG2 was significantly reduced in the HUA group compared to the NC group. However, treatment with JST-1 and JST-2 reversed this decline, restoring ABCG2 expression (*p* < 0.001). Additionally, all three iridoid glycoside treatment groups counteracted the downregulation of OAT1 and OAT3 protein expression observed in the HUA group.

### 2.7. Results of Molecular Docking

**Figure 8:**
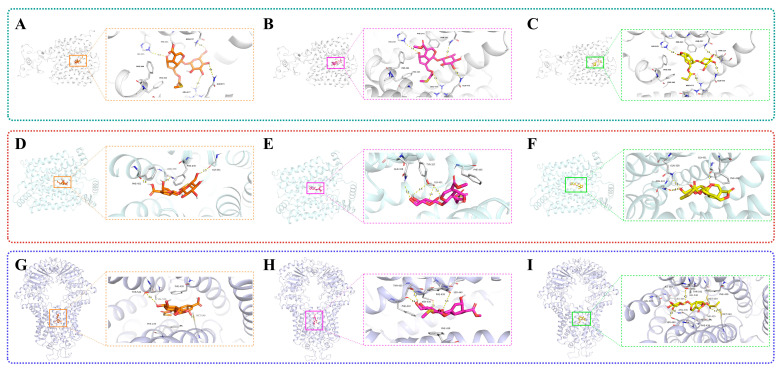
Compounds JST-1, -2, and -3 were modeled into URAT1 (PDB ID: 9JE0). (**A**–**C**) Compounds JST-1, -2, and -3 were modeled into GLUT9 (PDB ID: 8Y66). (**D**–**F**) Compounds JST-1, -2, and -3 were modeled into ABCG2 (PDB ID: 6VXJ). (**G**–**I**) The yellow, pink, and green boxes represent the binding sites for JST-1, JST-2, and JST-3, respectively. The ligands and key residues are displayed as a stick representation, while hydrogen bonds are illustrated as yellow dashed lines.

The binding scores of the ligand compound molecules obtained from molecular docking are presented in Table 1. Molecular docking analysis in this study revealed higher binding affinities for the selected compounds to URAT1, GLUT9, and ABCG2. The visualization of ligand–protein interactive binding was employed to evaluate the formation and stabilization of chemical bonds. In Figure 8, the dashed line illustrates the occurrence of interactive bonds.

For the URAT1 protein target (Figure 8A–C), the results showed that the ligand compound JST-3 possessed the strongest binding score of −8.0 kcal mol^−1^, followed by ligand JST-1 with −7.5 kcal mol^–1^ and ligand JST-2 with −7.4 kcal mol^−1^. Figure 8A shows that the amino acid residues of HIS-254, ARG-477, GLN-473, THR217, and ASN-237 constituted the binding site for JST-3, through hydrogen bonds, as well as the amino acid residues of PHE-241, PHE-364, and PHE-365 which have hydrophobic interactions. Figure 8B indicates that amino acid residues HIS-254, ARG-477, GLN-473, and ASN-237 were the binding sites for JST-1 and URAT1. Figure 8C reveals that JST-2 was combined with URAT1 by amino acid residues HIS-254, ARG-477, GLN-473, and ASN-237.

These results showed that JST-1, -2, and -3 had a potentially high binding affinity for GLUT9 (Figure 8D–F). Figure 8D demonstrates that the amino acid residues of GLY-431, ASN-458, PHE-451, and PHE-435 serve as the binding sites for JST-1 and GLUT9. Figure 8E shows that amino acid residues GLY-431, GLN-328, TYR-327, and PHE-435 are the binding sites for JST-2 and GLUT9. Figure 8F demonstrates that JST-3 interacted with GLUT9 through the amino acid residues GLY-431, GLN-328, ASN-333, and PHE-435.

The findings indicated that JST-1, -2, and -3 may interact with ABCG2 (Figure 8G–I). Figure 8G shows that amino acid residues THR-542’, MET-549, PHE-439’, PHE-439, and VAL-546’ constituted the binding sites for JST-1 and ABCG2. Figure 8H demonstrates that amino acid residues ASN-436’, PHE-432’, THR-435’, PHE-439’, and PHE-439 were the binding sites for JST-2 and ABCG2. Figure 8I illustrates that JST-3 interacts with ABCG2 through amino acid residues MET-549’, VAL-546’, THR-542’, LEU405’, MET-549, ASN-436, SER-440, PHE-439’, PHE-439, ILE-543’, and THR-435. These bound interrelations may have resulted from hydrogen bonds, hydrophobic interactions, and P-π interactions.

## 3. Discussion

The global prevalence of hyperuricemia is increasing, and the age distribution of cases shows a trend toward younger individuals [37]. Hyperuricemia, defined by elevated levels of serum urate, is associated with a range of comorbidities, including gout [38], renal disorders [39], cardiovascular diseases [40], metabolic syndrome [41], diabetes [42,43], etc.

*Paederia foetida* Linn. is a dual-purpose medicinal and edible plant, which has recently generated much interest due to its potential role in the development of drugs [44]. The main chemical constituents of this plant are iridoid glycosides containing sulfur [45,46]. Therefore, it exhibits a distinct characteristic of emitting a strong, sulfurous odor when its leaves or stems are crushed or bruised [47]. Sulfur-containing natural products exhibit a wide variety of forms due to the multiple valence states of sulfur, ranging from S^2−^ to S^6+^ [48]. As expected, the thioesters in the compound can form a binding interaction with the amino acid residue ARG477 in the URAT1 protein. The thioesters in the JST-2 compound establish a binding interaction with the A-chain amino acid residue PHE439 in the ABCG protein. Similarly, the thioesters in the JST-3 compound interact with the amino acid residue MET549 located in the A chain of the ABCG2 protein. Their complex structures and diverse biological activities have drawn considerable attention from chemists worldwide.

This is the first study to evaluate the alleviating effects of three iridoid glycosides obtained from *Paederia foetida* Linn. on HUA by using a rat model induced by PO combined with adenine. Potassium oxonate, a selective and competitive inhibitor of uricase, inhibits the activity of hepatic uricase and induces hyperuricemia in rodents [49]. Adenine promotes the synthesis of uric acid, and the dietary consumption of high-purine foods can also lead to transient uricosuria, which may play a significant role in mediating kidney injury [50,51]. The rat model is characterized by long-term hyperuricemia, severe glomerular sclerosis, renal interstitial fibrosis, and renal dysfunction, which more closely mimic the clinical condition [52].

Following a single-dose administration, JST-1, -2, and -3 differentially reduced serum UA levels, with JST-3 demonstrating the most pronounced reduction, consistent with its acute hypouricemic efficacy. In contrast to the AP group, which significantly decreased both serum and urinary uric acid levels, the three iridoid glycosides failed to reduce urinary uric acid levels. This suggests that their hypouricemic mechanism may involve promoting uric acid clearance rather than inhibiting xanthine oxidase. Notably, JST-3 exhibited a particularly pronounced reduction in urine volume, which may indicate potential renal side effects associated with this compound. Additionally, after one week of treatment, the urinary nitrogen levels in the JST-3 group were abnormally elevated, suggesting possible nephrotoxicity. However, the precise mechanism underlying these observations remains unclear and requires further investigation. Particular attention to serum markers (e.g., ALT, AST) and a histological examination of organs other than the kidneys after treatment with JST-3 will enhance our toxicity assessment.

It has been reported that kidney injury is characterized by a marked decline in kidney function and the accumulation of metabolic waste products, such as creatinine and blood urea nitrogen (BUN) [53]. Uric acid and creatinine are the end products of purine metabolism and muscle breakdown [54], respectively, and serve as critical indicators for assessing renal function. The results demonstrate that the levels of BUN and CRE of the HUA group exhibited markedly elevated relative to those in the NC group, indicating a substantial decline in renal function within the HUA group. This observation was further validated through H&E staining analysis. Moreover, CRE clearance represents a key parameter for evaluating renal filtration efficiency, a common alternative assay for the measured glomerular filtration rate (mGFR) [55], which provides a quantitative evaluation of renal function. The reduction in the glomerular filtration rate (GFR) associated with renal impairment leads to an adaptive increase in the fractional excretion of urate [56]. The experimental outcomes suggest that treatment with three iridoid glycosides significantly ameliorated hyperuricemia-induced kidney injury in rats, and JST-1 and JST-2 can not only reduce the levels of BUN and serum CRE but also exert significant renal protective effects by reducing renal excretion fraction, enhancing the 24 h CCR and 24 h CUA.

In our study, we were the first to demonstrate that JST-1, -2, and -3 interventions inhibited the protein expression of URAT1 and GLUT9 while upregulating the protein expression of ABCG2, OAT1, and OAT3, as determined by immunofluorescence analysis. Hyperuricemia has been associated with an increased risk of progression in chronic kidney disease [57]. The processes of uric acid reabsorption and secretion are mediated by a series of membrane transporters, including urate transporter 1 (URAT1) [58], GLUT9, ABCG2, OAT1, and OAT3. URAT1 serves as the primary transporter for urate absorption and functions as a therapeutic target of anti-hyperuricemia drugs [59]. It is increasingly acknowledged that disturbances in urate transport are pivotal in the pathogenesis of diseases associated with hyperuricemia. As two-thirds of human urate excretion takes place via the kidneys, reduced kidney function is closely associated with hyperuricemia. Our previous study confirmed that the molecular docking calculations revealed higher binding affinities of three iridoid glycosides to URAT1, GLUT9, and ABCG2. The binding energy reflects the affinity between the compounds and the target proteins. The simulated docking affinity values indicate that JST-3 exhibits the highest affinity for URAT1 (−8.0 kcal/mol), JST-1 shows the highest affinity for GLUT9 (−7.7 kcal/mol), and JST-3 demonstrates the highest affinity for ABCG2 (−7.8 kcal/mol). The three iridoid glycosides may act through distinct molecular targets, potentially related to their unique chemical structures, warranting further mechanistic investigation.

Our current research has certain limitations. Specifically, we did not conduct in vivo absorption, distribution, metabolism, excretion (ADME) studies on JST-1, -2, and -3. However, recent studies have reported the pharmacokinetic (PK) behavior of JST-1, -2, and -3 in rats following oral gavage and the intravenous injection of *Paederia scandens* extract, at single doses of 3 g/kg and 50 mg/kg, respectively. After oral administration in rats, JST-2 and JST-3 were absorbed rapidly, with Tmax values between 0.29 and 0.33 h. In contrast, JST-1 absorption was about 1.5 h slower, exhibiting a Tmax of 1.69 h. After intravenous injection in rats, the t_1/2_ of JST-1, -2, and -3 ranged from 1.21 to 3.16 h. The oral bioavailability of JST-1, -2, and -3 was 3.36 ± 0.14%, 2.00 ± 0.62%, and 1.84 ± 0.60%, respectively [60].

According to Lipinski’s Rule of Five, the physicochemical properties of JST-1, -2, and -3 exhibit high similarity to other iridoid glycosides, such as geniposidic acid, geniposide [61], monotropein [62], asperulosidic acid, asperuloside, and loganin [63]. The absorption characteristics of these compounds suggest that they are primarily absorbed via passive diffusion and are potential substrates of P-glycoprotein. These compounds are well distributed in the liver, kidneys, and intestines, which are considered target organs for urate-lowering therapy [64]. After oral administration, iridoid glycosides are metabolized via phase I and II pathways, including the hydrolysis of the glycosidic bond, demethylation, methylation, cysteine conjugation, glycosylation, and glucuronide conjugation [65]. Meanwhile, iridoid glycosides can be extensively hydrolyzed to aglycones in the intestine. In addition, iridoid glycosides are excreted via urine [61]. In further work, we will focus on the ADME of the three iridoid glycosides when giving single compounds or extracts to animals orally or intravenously.

## 4. Materials and Methods

### 4.1. Materials and Chemicals

Paederosidic acid (JST-1, purity ≥ 98%), paederosidic acid methyl ester (JST-2, purity ≥ 98%), and paederoside (JST-3, purity ≥ 95%) were extracted and purified from *Paederia foetida* Linn. Allopurinol (AP, Specifications: 100 mg, Lot: 110802) was obtained from Shanghai Sine Wanxiang Pharmaceuticals Co., Ltd. (Shanghai, China). Benzbromarone (BM, Specifications: 50 mg, Lot: H20171301) was provided by Excella GmbH & Co. KG (Feucht, Germany). Potassium oxonate (PO, Lot: P1275208) was provided by Adamas Reagent Co., Ltd. (Shanghai, China). Adenine (Ade, Lot: X01012MA13) was purchased from Shanghai Yuanye Bio-Technology Co., Ltd. (Shanghai, China). D101 resin was purchased from Cangzhou Baoen Adsorption Material Technology Co., Ltd. (Cangzhou, China).

The assay kits for uric acid (UA, Lot: G884, B870), blood urea nitrogen (BUN, Lot: B868, A868), and creatinine (CRE, Lot: E843) were provided by Shino Clinical Diagnostic Products Co., Ltd. (Chiyoda City, Japan). The assay kit for URAT1 was provided by Wuhan Cloud-Clone Corp. (Wuhan, China). Antibodies for URAT1, GLUT9, ABCG2, OAT1, and OAT3 (Table 2) were obtained from Proteintech Group, Inc. (Wuhan, China), while that for GAPDH was obtained from Affinity Biosciences (Changzhou, China).

### 4.2. Extraction and Purification

**Figure 9:**
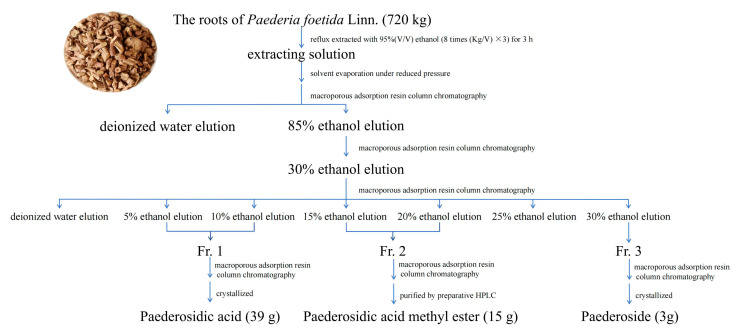
The preparation process of three iridoid glycosides from *Paederia foetida* Linn.

**Figure 10:**
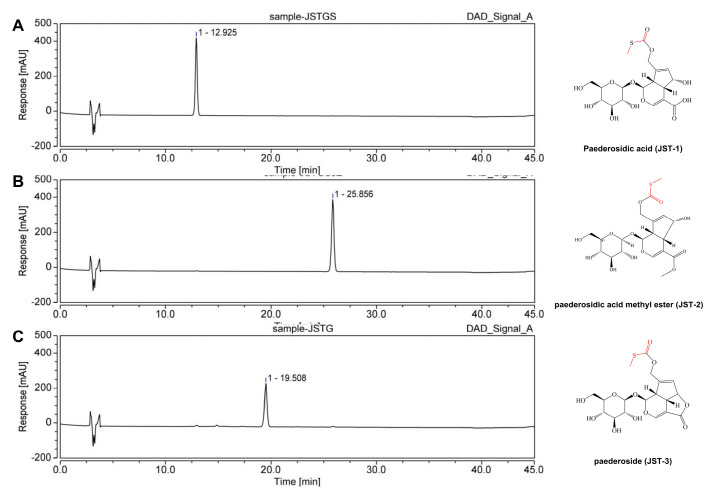
The structure and HPLC chromatogram of three iridoid glycosides. (**A**) Paederosidic acid; (**B**) paederosidic acid methyl ester; (**C**) paederoside. The red bond is a sulfur-containing structural unit.

The roots of *Paederia foetida* Linn. (720 kg) were reflux-extracted with 95% (*v*/*v*) ethanol (8 times (Kg/V) × 3) for 3 h. The extracting solution was evaporated under reduced pressure to afford a residue, which was then suspended in water and purified by macroporous adsorption resin (Model: D101) column chromatography eluted with water (2 column volume, CV) and 85% ethanol (5 CV) subsequently. The column volume is 24 L, and the ratio of column diameter to height is 1:8. The sample loading flow rate is 1 column volume per hour, and the elution flow rate is 5 column volumes per hour. The 85% ethanol fraction was further purified by column chromatography eluted with water (2 CV) and 30% (*v*/*v*) ethanol (8 CV). The 30% ethanol eluent was evaporated under reduced pressure to afford a crude extract (12.24 kg). A portion of crude extract (200 g) was subjected to macroporous adsorption resin column chromatography (column volume is 3 L, column diameter is 10 cm, column height is 120 cm) eluted with deionized water and ethanol–H_2_O (5%, 10%, 15%, 20%, 25%, and 30% ethanol successively, with 5 CV each). After solvent evaporation under reduced pressure, three semidried fractions were obtained: Fr.1 (combine eluents with 5% and 10% ethanol), Fr.2 (combine eluents with 15% and 20% ethanol), and Fr.3 (30% ethanol eluent solution). Fr.1 was further purified by macroporous adsorption resin column chromatography (column size 3 L), and 5% ethanol (25 L) eluate was collected to afford a residue, which was dissolved in methanol, then crystallized after filtration. The crystal is paederosidic acid (JST-1, 39 g), which was determined to be 98.06% by the HPLC analysis external standard method. Fr. 3 was treated in the same way as Fr.1, and 15% ethanol eluate (25 L) was collected, dissolved in methanol, then crystallized; finally, paederosidic acid methyl ester (JST-2, 15 g) was obtained, which was determined to be 99.26% by HPLC analysis. Fr. 2 was purified like Fr.1, and 15% ethanol (25 L) eluate was collected, which was purified by preparative HPLC (SepaFlash semi-spherical C18, 50 μm, 90 Å, 100 g (Santai Technologies, Inc., Changzhou, China); mobile phase, MeOH/H_2_O gradient; detection at 230 nm) to afford paederoside (JST-3, 3 g), and its purity was 96.83%. The preparation process of three iridoid glycosides is shown in Figure 9. The structure and HPLC chromatograms of three iridoid glycosides are shown in Figure 10.

### 4.3. Experimental Animals

A total of 96 male Sprague-Dawley (SD) rats, aged 5 weeks and weighing 200 ± 20 g, were purchased from Shanghai SLAC Laboratory Animal Co., Ltd. (Shanghai, China) (Certificate No.: SCXK (Hu) 2018-0006). The SD rats were housed and maintained at the Shanghai Institute of Pharmaceutical Industry (Shanghai, China) (Certificate No.: SYXK (Hu) 2019-0027) under specialized pathogen-free (SPF) conditions. These conditions included a controlled temperature of 25 ± 2 °C, humidity ranging from 50% to 75%, and a 12 h light/dark cycle. The rats underwent a one-week acclimatization period during which they had free access to food and water under the aforementioned conditions.

### 4.4. Animal Protocol

#### 4.4.1. Acute Hyperuricemia

After one week of adaptive feeding, 54 SD rats were randomly divided into 9 groups (n = 6/group) as follows: the normal control group (NC), acute hyperuricemia group (HUA) [66], allopurinol positive control group (50 mg/kg) (AP), low-dose three iridoid glycoside groups (20 mg/kg for JST-1, -2, and -3), and high-dose three iridoid glycoside groups (40 mg/kg for JST-1, -2, and -3). After grouping, except for the NC group, all other groups of rats were given potassium oxonate suspension (300 mg/kg). One hour later, rats in the AP and three iridoid glycoside groups were administered drugs by gavage. One hour after the final administration, blood samples were collected via the retro-orbital route for the detection of uric acid levels. Changes in serum uric acid content were quantified using commercially available kits.

#### 4.4.2. Hyperuricemia-Induced Kidney Injury

**Figure 11:**
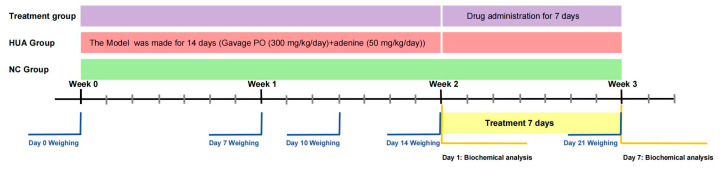
Flowchart of experimental treatment of hyperuricemia-induced kidney injury.

After one week of adaptive feeding, 42 SD rats were randomly divided into 7 groups (n = 6/group): the normal control group (NC), hyperuricemia group (HUA), allopurinol positive control group (20 mg/kg/day) (AP), benzbromarone positive control group (50 mg/kg/day) (BM), and three iridoid glycoside groups (40 mg/kg/day for JST-1, -2, and -3 groups). Rats in the NC group received an equal volume of saline, whereas those in the other groups were administered intraperitoneal PO (300 mg/kg/day) and intragastrical adenine (50 mg/kg/day) for 14 days [67]. Subsequently, rats in the pharmaceutical intervention groups were given their respective drug doses once daily for 7 days, while those in the NC and HUA groups were administered saline via gavage for 7 days. The body weights of all rats were recorded throughout the experiment (Figure 11).

On the first and seventh days of administration, one hour after drug/saline administration, blood was collected from the retro-orbital route, and 24 h urine was collected from rats using metabolic cages. Uric acid, creatinine, and urea nitrogen indicators were measured in serum and urine, and the creatinine clearance rate, uric acid clearance rate, and FEUA were calculated. The rats were sacrificed after the last blood sampling according to ethical animal euthanasia guidelines. The kidneys of the rats were removed, and the total weight of the kidneys was recorded.

### 4.5. Biochemical Analysis

Following 60 min of coagulation, blood samples were centrifuged at 10,000 rpm for 10 min at 4 °C to obtain serum. Urine supernatants were collected after the centrifugation of the corresponding urine samples. The levels of serum urate (SU), urinary uric acid (UA), serum creatinine (CRE), urinary creatinine (CRE), and blood urea nitrogen (BUN) were measured according to the kit instructions. Additionally, 24 h urine volume (24 h UV) was recorded.

The fractional excretion of uric acid (FEUA) was expressed as follows:

$$\mathrm{FEUA}\;\%=\;\frac{Serum\;CRE\;\times \;Urine\;UA}{Serum\;Urate\;\times \;Urine\;CRE}\times 100\%$$

The creatinine clearance rate (CCR) and uric acid clearance rate (CUA) were expressed as follows:

$$24\;h\;\mathrm{CCR}\;=\;\frac{Urine\;CRE\;\times \;24\;h\;UV}{Serum\;CRE\;\times \;24\;\times \;60}$$

$$24\;h\;\mathrm{CUA}=\frac{Urine\;UA\times 24\;h\;UV}{Serum\;Urate\times 24\times 60}$$

### 4.6. Organ Coefficient Determination

The kidneys of the rats were collected post-euthanasia. After rinsing with normal saline, the organs were dried using standard filter paper and subsequently weighed. The organ coefficient was calculated as the ratio of the organ weight to the body weight.

### 4.7. Hematoxylin and Eosin (H&E) Staining Analysis

The kidney tissues were fixed in 4% paraformaldehyde for 24 h, followed by dehydration, wax infiltration, embedding, and sectioning at a thickness of 4 μm for hematoxylin and eosin (H&E) staining. The tissue sections were visualized and photographed under a NIKON Eclipse Ci microscope (Tokyo, Japan) equipped with an imaging system (NIKON digital sight DS-FI2, Tokyo, Japan) at 200× magnification.

### 4.8. Immunofluorescence (IF) Analysis

The paraffin sections were baked at 62 °C for 1 h and deparaffinized by using xylene/anhydrous ethanol. Then, the sections were rehydrated through a graded series of ethanol solutions. Following antigen retrieval and cooling, the sections were rinsed three times with citrate-buffered saline (pH 6.0, containing 3 g/L trisodium citrate and 0.37 g/L citric acid). Subsequently, bovine serum albumin (BSA) was applied to the tissue sections and incubated for 30 min. After drying by rotation, the appropriate primary antibodies (URAT1, GLUT9, ABCG2, OAT1, and OAT3) were added, followed by overnight incubation at 4 °C. The next day, the paraffin sections were washed three times and then incubated with HRP-labeled secondary antibodies matching the primary antibodies for 60 min. A DAPI stain was subsequently applied to the tissue sections and incubated for 10 min in the dark. A self-quenching agent was added for 5 min, followed by rinsing with water for 10 min after drying. Finally, the sections were sealed with an anti-fluorescence quencher and photographed using a microscope slide scanner (Nikon Eclipse Ci-L; Nikon, Tokyo, Japan). The immunofluorescence-positive area was quantified by thresholding fluorescence images to distinguish specific signals from background. The area of positive staining was normalized to the total tissue area and expressed as a percentage. Statistical analysis was performed to compare the fluorescence-positive area between experimental groups.

### 4.9. Molecular Docking

All chemical 3D structures of compounds were retrieved from the PUBCHEM database and saved in sdf format. The 3D crystal structure of the target protein was obtained from the RCSB Protein Data Bank (https://www.rcsb.org/). The protein preparation wizard tool was employed to remove water molecules, ions, and attached ligands, with optimization and restrained minimization performed using PyMOL molecular visualization software Version 2.3.0. Using AutoDockTools-1.5.7, polar hydrogen atoms were added to the protein, Gasteiger charges were calculated, and the structure was saved in pdbqt format. For the ligands’ 3D structures, hydrogen atoms were added, Gasteiger charges were calculated, root atoms were selected, rotatable bonds were set, and the structure was saved in pdbqt format using AutoDockTools-1.5.7. Docking and interaction binding evaluations were carried out using AutoDock Vina 1.1.2 with a grid box size-adjusted at 30 Å × 40 Å × 30 Å for URAT1, 30 Å × 30 Å × 30 Å for ABCG2, and 32 Å × 40 Å × 32 Å for GLUT9. The exhaustiveness parameter was set to 50. After docking, the most reasonable binding modes were selected based on binding affinity and interaction patterns. PyMOL was subsequently used for molecular visualization and a detailed analysis of the protein–ligand interactions.

### 4.10. Statistical Analysis

All data were expressed as mean ± SEM and were analyzed with SPSS v. 29.0 for Windows (IBM Corp., Armonk, NY, USA). Differences among groups were analyzed using One-way ANOVA, followed by Least Significant Difference multiple comparison test. *p* < 0.05 was considered as significant difference. All figures were drawn by Prism 6 software (GraphPad, San Diego, CA, USA).

## 5. Conclusions

Our study provides evidence that three unique sulfur-containing iridoid glycosides isolated from *Paederia foetida* Linn. significantly alleviate hyperuricemia and hyperuricemia-induced renal injury in a rat model induced by potassium oxonate and adenine. The results showed that the intervention of all three iridoid glycosides reduced several HUA-related markers, including serum BUN and CRE production.

The results of this study suggested that these compounds exert their hypouricemic and renoprotective effects by modulating the expression of key uric acid transporters, thereby enhancing uric acid excretion. The underlying mechanisms are proposed to involve the following pathways: the inhibition of uric acid reabsorption, the promotion of uric acid secretion, and the improvement of renal function. These findings underscore the therapeutic potential of these iridoid glycosides in the management of hyperuricemia and its associated complications. Future research will focus on identifying the ADME and precise molecular targets of these compounds, elucidating their structure–activity relationships, and optimizing their therapeutic efficacy to fully clarify their mechanisms of action.

## Figures and Tables

**Table 1:** The computational docking results demonstrating the binding interactions between three structurally distinct iridoid glycosides and key urate-regulating proteins (URAT1, GLUT9, ABCG2).

Target		Ligand Compound		Ligand Compound	Binding Score (kcal mol^−1^)
Target (PDB ID)	Target Structure	Ligand Compound	3D Structure		
URAT1 (9JE0)	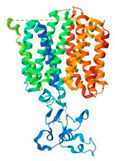	Paederosidic acid (JST-1)	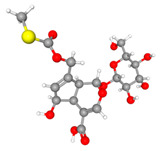	JST-1	−7.5
				JST-2	−7.4
				JST-3	−8.0
GLUT9 (8Y66)	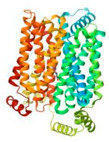	Paederosidic acid methyl ester (JST-2)	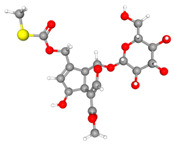	JST-1	−7.7
				JST-2	−7.3
				JST-3	−7.6
ABCG2 (6VXJ)	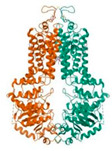	Paederoside (JST-3)	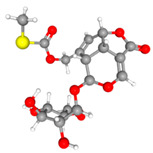	JST-1	−7.0
				JST-2	−7.2
				JST-3	−7.8

**Table 2:** Antibodies for immunofluorescence analysis.

Antibody	Company	Catalog Number	Dilution Ratio
BCRP/ABCG2 Polyclonal antibody	Proteintech	27286-1-AP	1:400
URAT1 Polyclonal antibody		14937-1-AP	1:300
SLC2A9 Polyclonal antibody		26486-1-AP	1:200
OAT1 Polyclonal antibody		26574-1-AP	1:400
SLC22A8 Antibody	Abmart (Shanghai, China)	TD7196F	1:400
Goat Anti-Rabbit IgG H&L (HRP)	Abcam (Cambridge, UK)	Ab6721	1:1000

## Data Availability

The original contributions presented in this study are included in the article/Supplementary Material. Further inquiries can be directed to the corresponding author.

## Supplementary Materials

The following supporting information can be downloaded at: https://www.mdpi.com/article/10.3390/molecules30153098/s1, Figure S1. The Chromatogram, Integration Results and UV-VIS spectrum of paederosidic acid (JST-1). Figure S2. The Chromatogram, Integration Results and UV-VIS spectrum of paederosidic acid methyl ester (JST-2). Figure S3. The Chromatogram, Integration Results and UV-VIS spectrum of paederoside (JST-3). Figure S4. 1H NMR spectrum of paederosidic acid (JST-1) (CDCl3, 600 MHz). Figure S5. 13C NMR spectrum of paederosidic acid (JST-1) (CDCl3, 600 MHz). Figure S6. 1H NMR spectrum of paederosidic acid methyl ester (JST-2) (CDCl3, 600 MHz). Figure S7. 13C NMR spectrum of paederosidic acid methyl ester (JST-2) (CDCl3, 600 MHz). Figure S8. 1H NMR spectrum of paederoside (JST-3) (CDCl3, 600 MHz). Figure S9. 13C NMR spectrum of paederoside (JST-3) (CDCl3, 600 MHz). Figure S10. The UPLC-Q-Tof-MS Chromatograms of Paederosidic acid (JST-1) on Negative Ionization Mode. A: UV chromatogram at 235nm; B: Total ion 31 chromatogram; C: Mass spectrum. Figure S11. The mass spectrum of Paederosidic acid (JST-1) on Positive Ionization Mode. Figure S12. TThe UPLC-Q-Tof-MS Chromatograms of Paederosidic acid methyl ester (JST-2) on Negative Ionization Mode. A: UV chromatogram at 235nm; 34 B: Total ion chromatogram; C: Mass spectrum. Figure S13. The mass spectrum of paederosidic acid methyl ester (JST-2) on Positive Ionization Mode. Figure S14. The UPLC-Q-Tof-MS Chromatograms of Paederoside (JST-3) on Negative Ionization Mode. A: UV chromatogram at 235nm; B: Total ion 37 chromatogram; C: Mass spectrum. Figure S15. The mass spectrum of paederoside (JST-3) on Positive Ionization Mode.

## Abbreviations

The following abbreviations are used in this manuscript:

HUA	Hyperuricemia
SD rat	Sprague-Dawley Rat
UA	Uric Acid
BUN	Blood Urea Nitrogen
UUN	Urine Urea Nitrogen
CRE	Creatinine
CCR	Creatinine Clearance Rate
CUA	Uric Acid Clearance Rate
FEUA	Fraction Excretion of Uric Acid
AP	Allopurinol
BM	Benzbromarone
PO	Potassium Oxonate
JST-1	Paederosidic Acid
JST-2	Paederosidic Acid Methyl Ester
JST-3	Paederoside
CB	Column Volume
URAT1	URAT1, Uric Acid Transporter 1
GLUT9	Glucose Transporter 9
ABCG2	ATP-Binding Cassette Transporter G2
OAT1	Organic Anion Transporter 1
OAT3	Organic Anion Transporter 3
ADME	Absorption, Distribution, Metabolism, Excretion
