# Supplementary material for: The Urate-Lowering Effects and Renal Protective Activity of Iridoid Glycosides from Paederia foetida in Rats with Hyperuricemia-Induced Kidney Injury: A Pharmacological and Molecular Docking Study
Source: Molecules. 2025 Jul 24;30(15):3098. doi: 10.3390/molecules30153098 (PMC12348965; doi:10.3390/molecules30153098)
Supplement: Supplementary file 1 [file molecules-30-03098-s001.zip › molecules-3735916-supplementary.pdf]

SUPPLEMENTARY MATERIAL

The Urate-Lowering Effects and Renal Protective Activity of Iridoid Glycosides from *Paederia foetida* in Rats with Hyperuricemia-Induced Kidney Injury: A Pharmacological and Molecular Docking Study

Haifeng Zhou [1,2], Xinyi Yue [2], Longhai Shen [2], Lifeng Wu [3], Xiaobo Li [1] and Tong Wu [2,*]

1 School of Pharmacy, Shanghai Jiao Tong University, Shanghai 200240, China; xfphoenix@163.com (H.Z.); xbli@sjtu.edu.cn (X.L.)
2 National Key Laboratory of Lead Druggability Research, Shanghai Institute of Pharmaceutical Industry, China State Institute of Pharmaceutical Industry, Shanghai 201203, China; eyeeye88@163.com (X.Y.); shenlh28@163.com (L.S.)
3 Ningo Dachang Pharmaceutical Co., Ltd., Ningbo 201203, China; foxwlf@hotmail.com
* Correspondence: wutong1@sinopharm.com; Tel.: +86-021-20572000 (ext. 3044)

Table of Contents

1. Supplementary high-performance liquid chromatography for three iridoid glycosides 3

1.1 Sample Preparation 3

1.2 Chromatography conditions 3

1.3 HPLC-analysis of three iridoid glycosides 3

Figure S1. The Chromatogram, Integration Results and UV-VIS spectrum of paederosidic acid (JST-1). 4

Figure S2. The Chromatogram, Integration Results and UV-VIS spectrum of paederosidic acid methyl ester (JST-2). 5

Figure S3. The Chromatogram, Integration Results and UV-VIS spectrum of paederoside (JST-3). 6

2. Supplementary ^{1}H-NMR and ^{13}C-NMR spectrum for three iridoid glycosides 7

Figure S4. ^{1}H NMR spectrum of paederosidic acid (JST-1) (CDCl3, 600 MHz). 7

Figure S5. ^{13}C NMR spectrum of paederosidic acid (JST-1) (CDCl3, 600 MHz). 8

Figure S6. ^{1}H NMR spectrum of paederosidic acid methyl ester (JST-2) (CDCl3, 600 MHz). 9

Figure S7. ^{13}C NMR spectrum of paederosidic acid methyl ester (JST-2) (CDCl3, 600 MHz). 10

Figure S8. ^{1}H NMR spectrum of paederoside (JST-3) (CDCl3, 600 MHz). 11

Figure S9. ^{13}C NMR spectrum of paederoside (JST-3) (CDCl3, 600 MHz). 12

3. Supplementary high-resolution mass spectrometry data for three iridoid glycosides 13

3.1 Sample Preparation 13

3.2 Chromatography and MS conditions 13

3.3 Q-Tof-MS Chromatograms 14

Figure S10 The UPLC-Q-Tof-MS Chromatograms of Paederosidic acid (JST-1) on Negative Ionization Mode. A: UV chromatogram at 235nm; B: Total ion chromatogram; C: Mass spectrum. 15

Figure S11 The mass spectrum of Paederosidic acid (JST-1) on Positive Ionization Mode. 16

Figure S12 TThe UPLC-Q-Tof-MS Chromatograms of Paederosidic acid methyl ester (JST-2) on Negative Ionization Mode. A: UV chromatogram at 235nm; B: Total ion chromatogram; C: Mass spectrum. 18

Figure S13 The mass spectrum of paederosidic acid methyl ester (JST-2) on Positive Ionization Mode. 19

Figure S14 The UPLC-Q-Tof-MS Chromatograms of Paederoside (JST-3) on Negative Ionization Mode. A: UV chromatogram at 235nm; B: Total ion chromatogram; C: Mass spectrum. 21

Figure S15 The mass spectrum of paederoside (JST-3) on Positive Ionization Mode. 22

1. Supplementary high-performance liquid chromatography for three iridoid glycosides

1.1 Sample Preparation

Paederosidic acid (JST-1), paederosidic acid methyl ester (JST-2) and paederoside (JST-3) were accurately weighed and dissolved in methanol to obtain sample solutions (0.2 mg/mL).

1.2 Chromatography conditions

HPLC analysis was performed on an Agilent 1260II chromatograph (Agilent Technologies, Santa Clara, CA, USA) equipped with a quaternary pump, an autosampler, and a photodiode array detector. The chromatographic conditions were as follows: an Agilent ZORBAX SB-C18 column (4.6 × 150 mm, 5 μm) (Agilent Technol.); The mobile phase consisted of water containing 0.1% formic acid as eluent A and acetonitrile as eluent B. The flow rate was set at a linear gradient as follows: 0–30 min, 15%→25% B; 30–35 min, 25%→15% B; 35–25 min, 15% B. The flow rate was 1 mL/min, the column temperature was 30℃, the injection volume was 10 μL, and the detection was performed at wavelengths 235 nm.

1.3 HPLC-analysis of three iridoid glycosides

Chromatogram

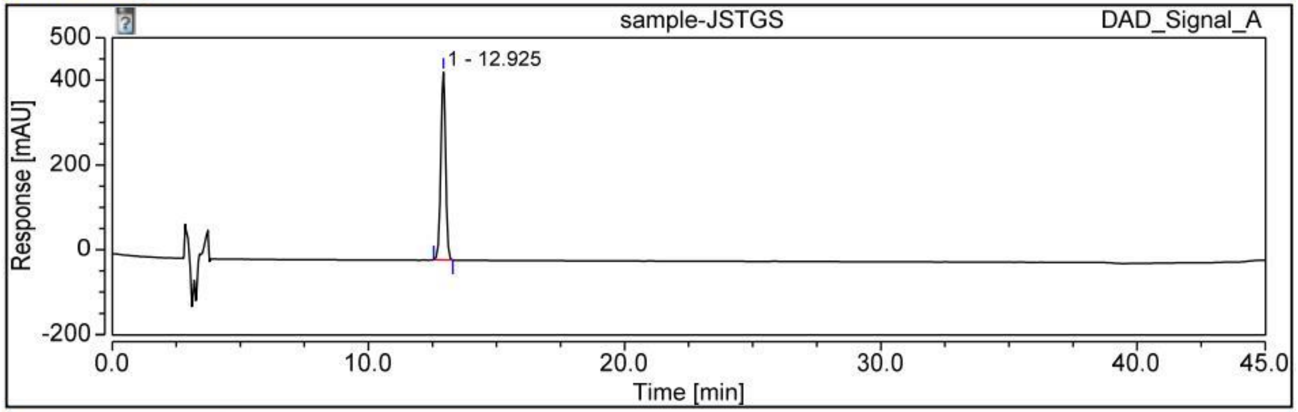

Integration Results

No.	Peak Name	Retention Time<br>min	Area<br>mAU*min	Height<br>mAU	Relative Area<br>%	Relative Height<br>%	Match
1		12.925	93.864	442.607	**100.00**	100.00	**1000**
**Total:**			**93.864**	**442.607**	**100.00**	**100.00**	

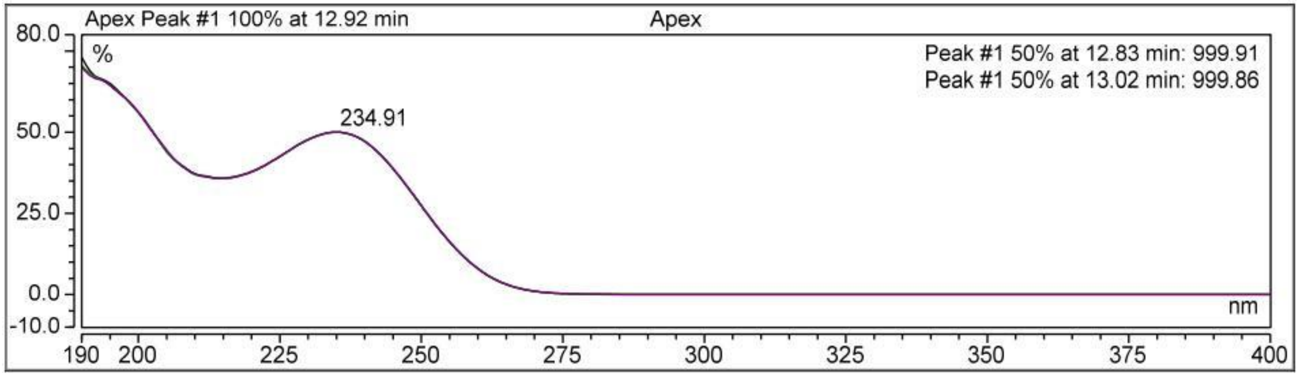

Figure S1. The Chromatogram, Integration Results and UV-VIS spectrum of paederosidic acid (JST-1).

Chromatogram

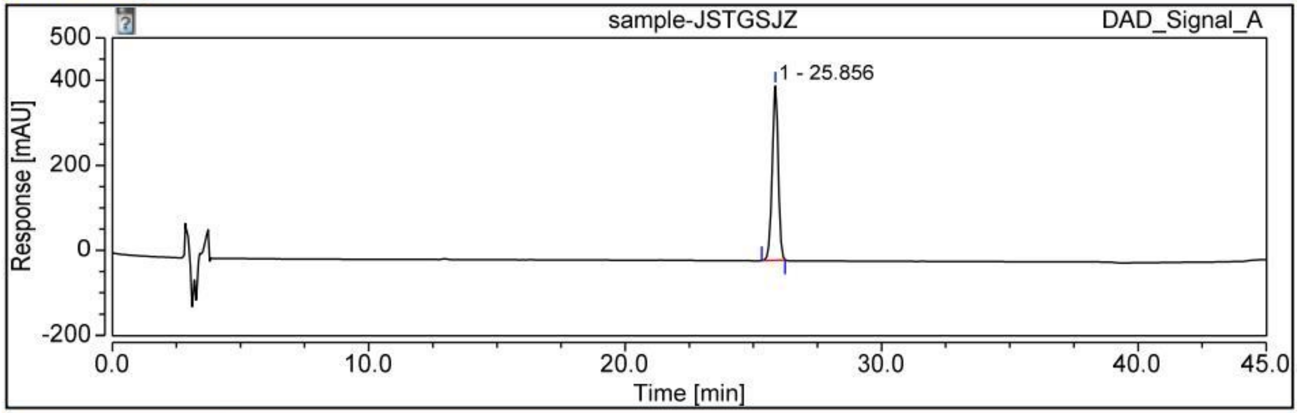

Integration Results

No.	Peak Name	Retention Time<br>min	Area<br>mAU*min	Height<br>mAU	Relative Area<br>%	Relative Height<br>%	Match
1		25.856	110.292	409.808	**100.00**	100.00	**1000**
**Total:**			**110.292**	**409.808**	**100.00**	**100.00**	

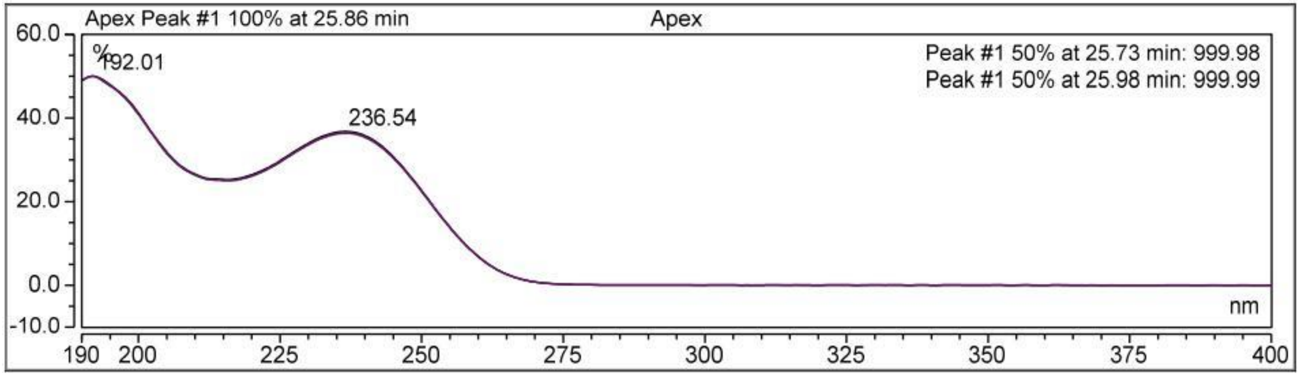

Figure S2. The Chromatogram, Integration Results and UV-VIS spectrum of paederosidic acid methyl ester (JST-2).

Chromatogram

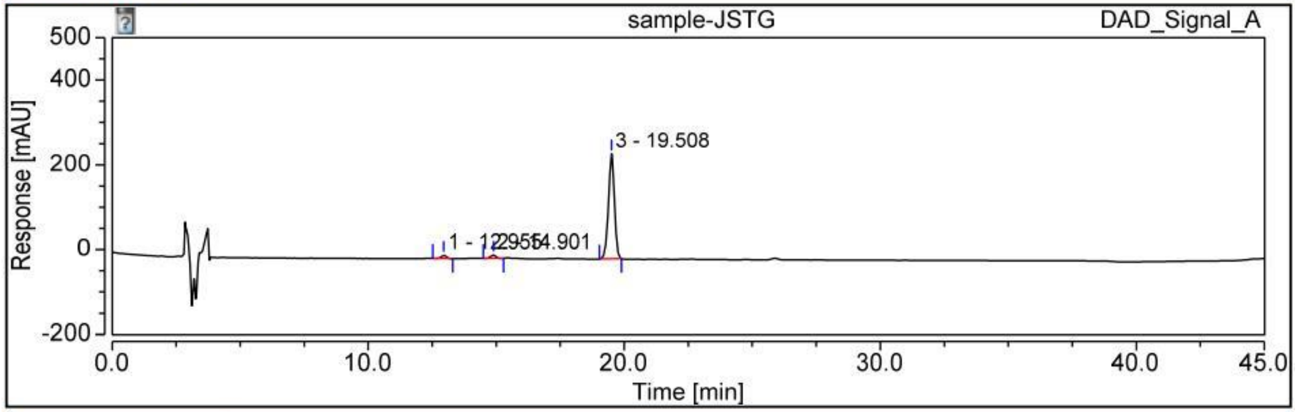

Integration Results

No.	Peak Name	Retention Time min	Area mAU*min	Height mAU	Relative Area %	Relative Height %	Match
1		12.955	1.645	7.573	**2.33**	2.88	**962**
2		14.901	1.700	7.718	**2.41**	2.93	**996**
3		19.508	67.164	247.682	**95.25**	94.19	**1000**
**Total:**			**70.510**	**262.973**	**100.00**	**100.00**	

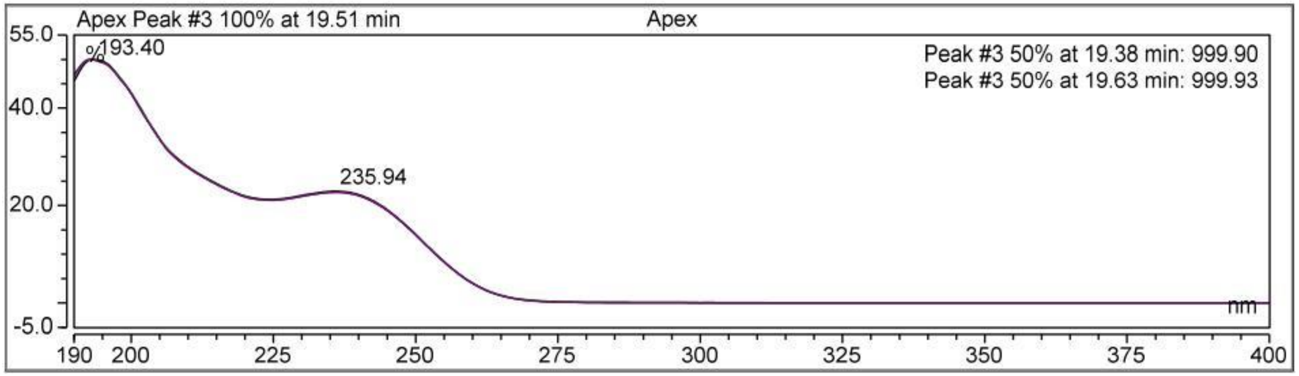

Figure S3. The Chromatogram, Integration Results and UV-VIS spectrum of paederoside (JST-3).

2. Supplementary ^{1}H-NMR and ^{13}C-NMR spectrum for three iridoid glycosides

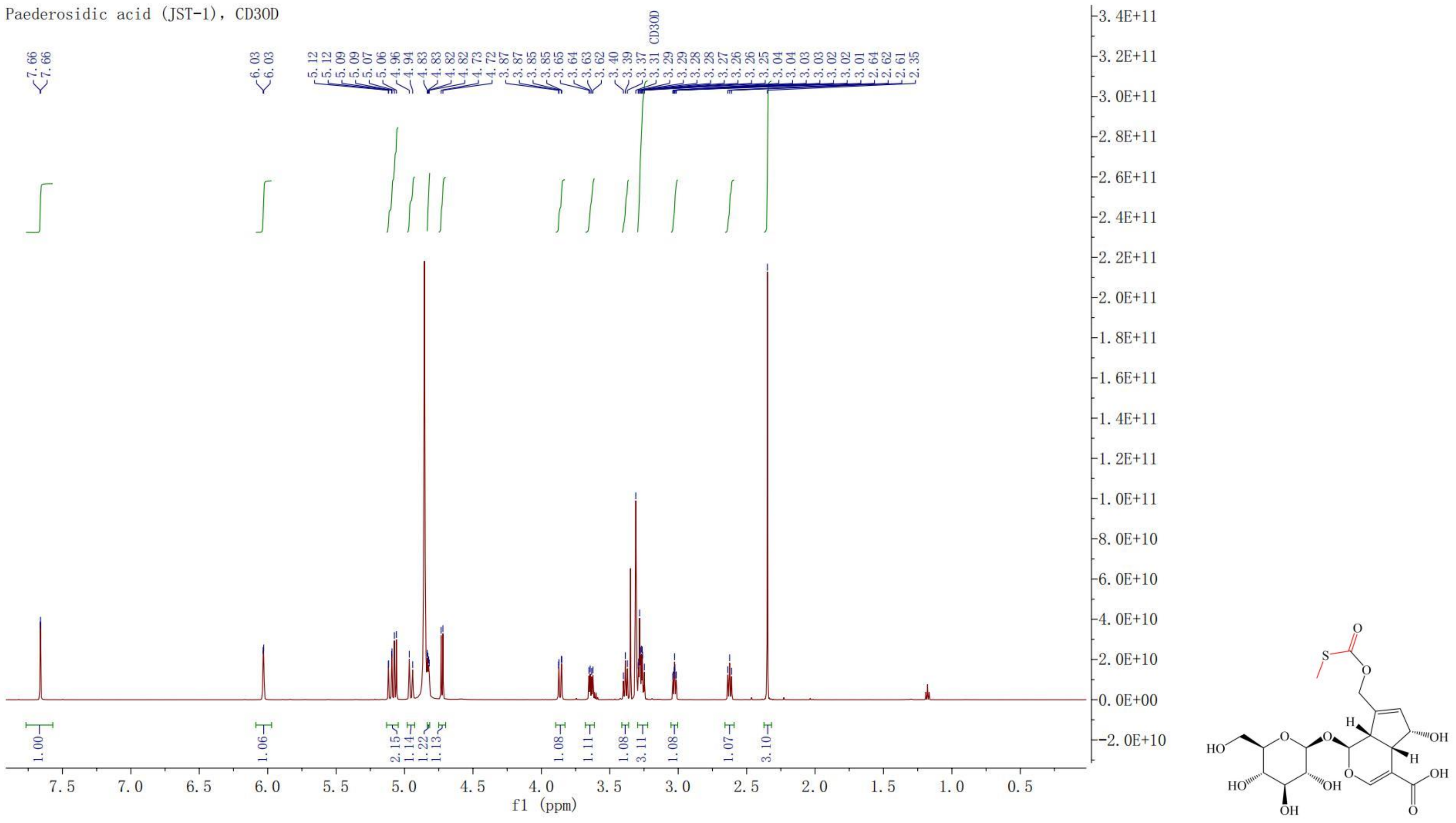

Figure S4. ^{1}H NMR spectrum of paederosidic acid (JST-1) (CDCl3, 600 MHz).

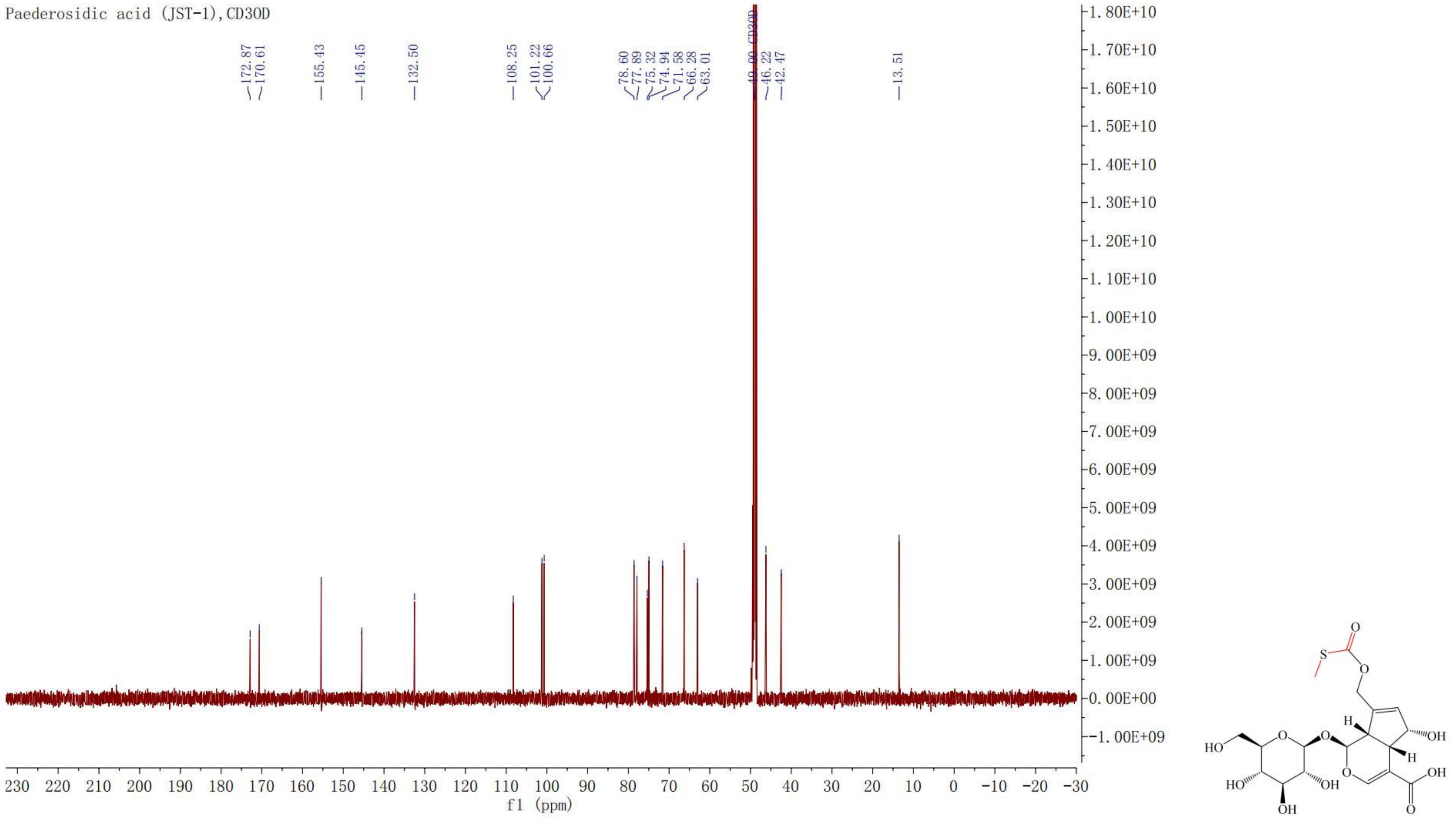

Figure S5. ^{13}C NMR spectrum of paederosidic acid (JST-1) (CDCl3, 600 MHz).

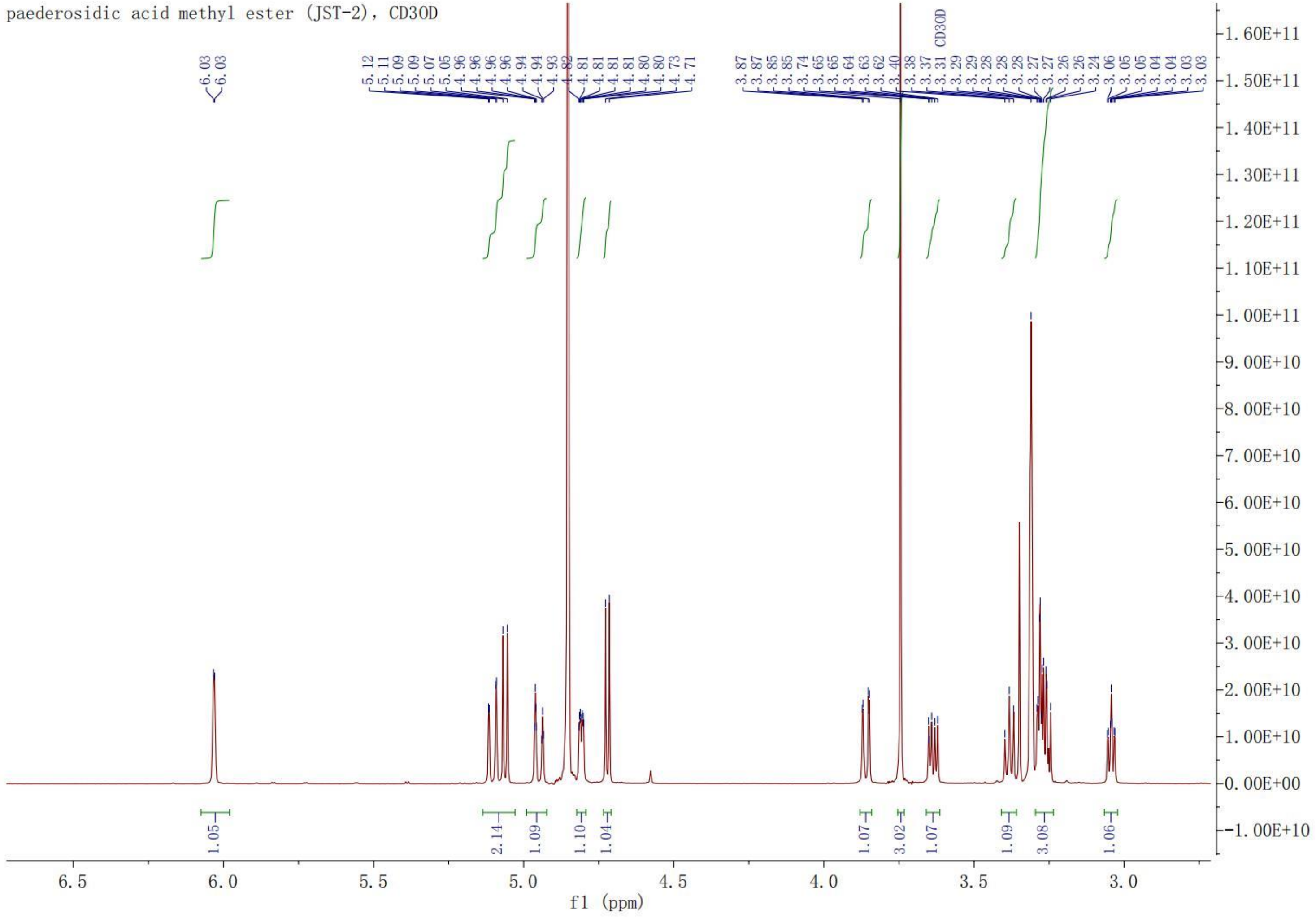

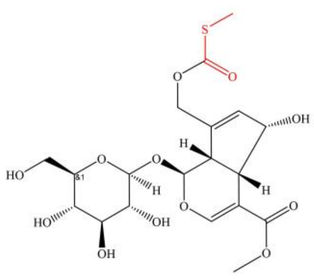

Figure S6. ^{1}H NMR spectrum of paederosidic acid methyl ester (JST-2) (CDCl3, 600 MHz).

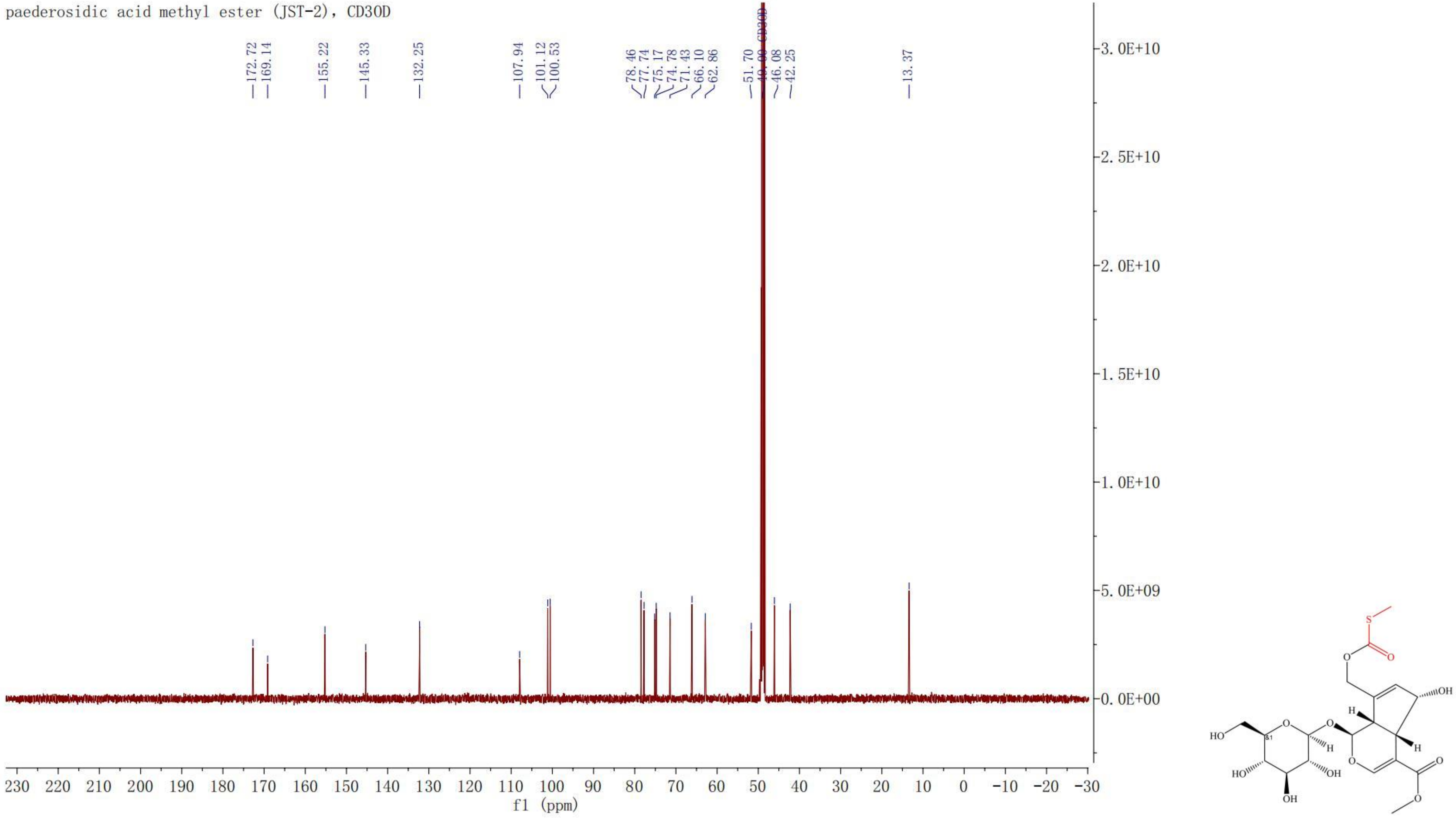

Figure S7. ^{13}C NMR spectrum of paederosidic acid methyl ester (JST-2) (CDCl3, 600 MHz).

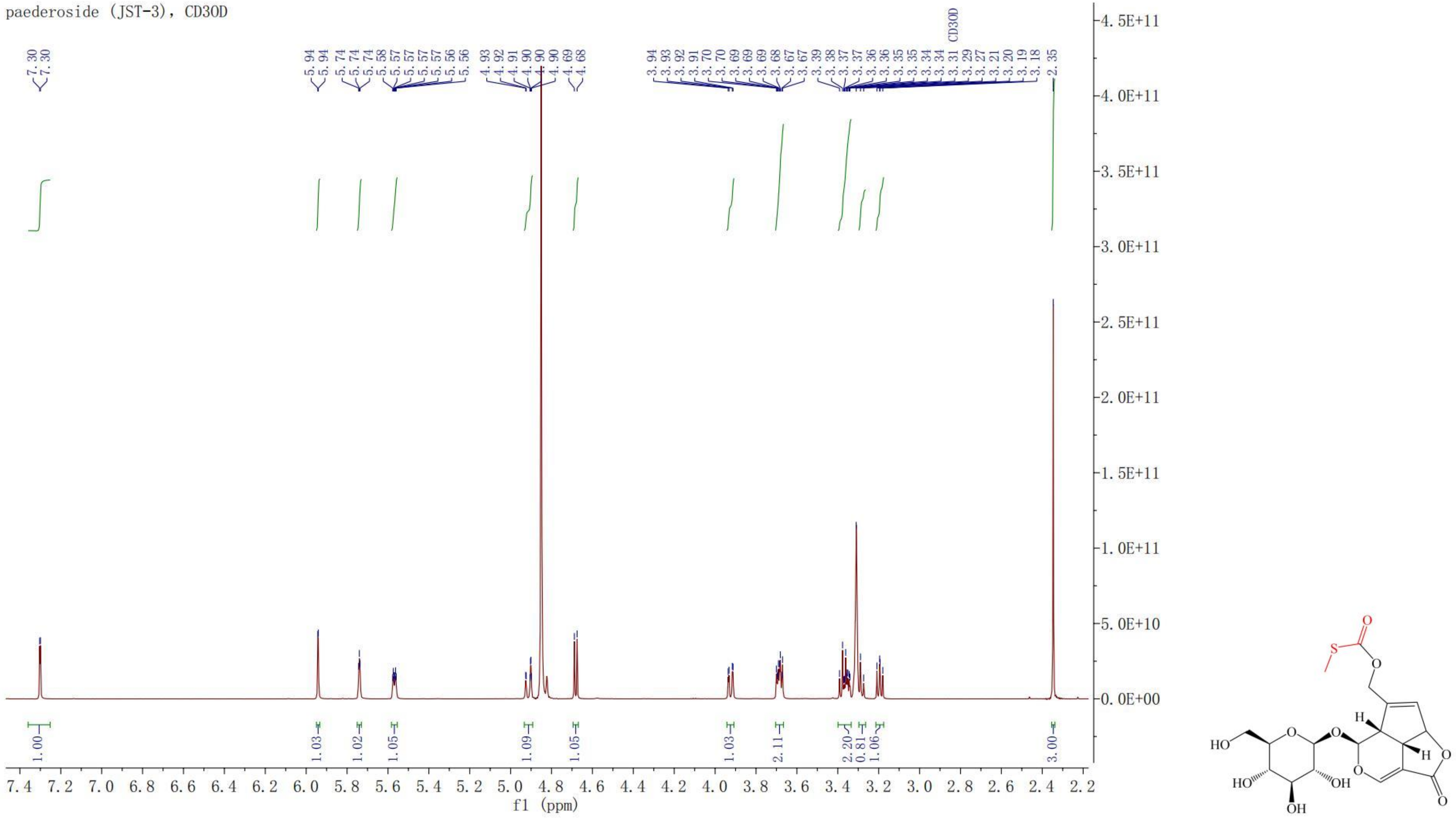

Figure S8. ^{1}H NMR spectrum of paederoside (JST-3) (CDCl3, 600 MHz).

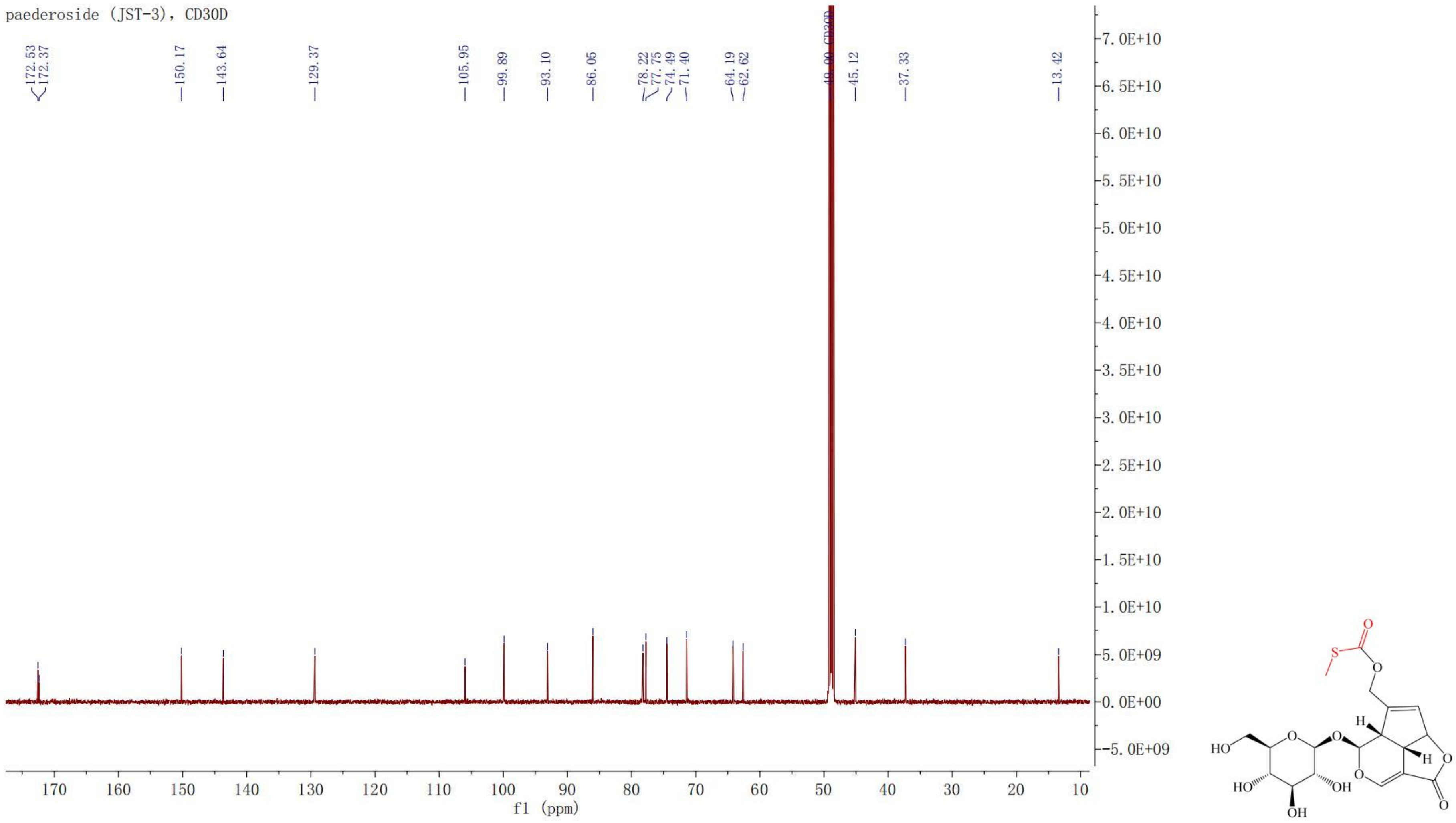

Figure S9. ^{13}C NMR spectrum of paederoside (JST-3) (CDCl3, 600 MHz).

3. Supplementary high-resolution mass spectrometry data for three iridoid glycosides

3.1 Sample Preparation

Paederosidic acid (JST-1), paederosidic acid methyl ester (JST-2) and paederoside (JST-3) were accurately weighed and dissolved in methanol to obtain sample solutions (0.2 mg/mL).

3.2 Chromatography and MS conditions

An Waters Acquity^{TM} UPLC system and a Xevo G2-XS Q-TOF MS system (Waters, Manchester, UK), equipped with an electrospray ionization (ESI) source were used for acquiring the MS data.

An ACQUITY UPLC^{TM} HSS C18 SB column (100 mm × 2.1 mm, 1.8 μm) was applied for chromatographic separation with a column temperature of 30°C with a flow rate of 0.35 mL/min. The mobile phase consisted of water containing 0.1% formic acid as eluent A and acetonitrile as eluent B. The flow rate was set at a linear gradient as follows: 0–5 min, 10%→15% B; 5–10 min, 15%→20% B; 10–11 min, 20%→10% B; 11–15 min, 10% B.

The conditions of the MS system were set as follows: Full-scan data were obtained from 200 to 1000 Da with a 0.3 s scan time, the desolvation gas (N2) flow rate was 600 L/h with a temperature of 250°C, the cone gas flow rate was 50 L/h, the source temperature was 120°C. The sample cone voltage was 40 V, the source offset voltage was 80 V and the capillary voltage was 3.5 kV. In the MS^{E} mode, the collision energy was 6 V for the low energy mode and 20 to 40 V for the high energy mode. Mass real-time correction was performed by infusing the solution of

leucine-enkephalin (0.2 ng/mL) at the rate of 10 μL/min via a lock spray interface to generate a reference ion for the negative ion mode (m/z 554.2615 Da $[M-H]^-$) and positive ion mode (m/z 556.2771 Da $[M+H]^+$) to ensure the accuracy of the MS analysis.

MS analysis in both positive and negative ion modes using an electrospray ionization (ESI). The Masslynx v4.1 (Waters, USA) was used for data acquisition.

3.3 Q-Tof-MS Chromatograms

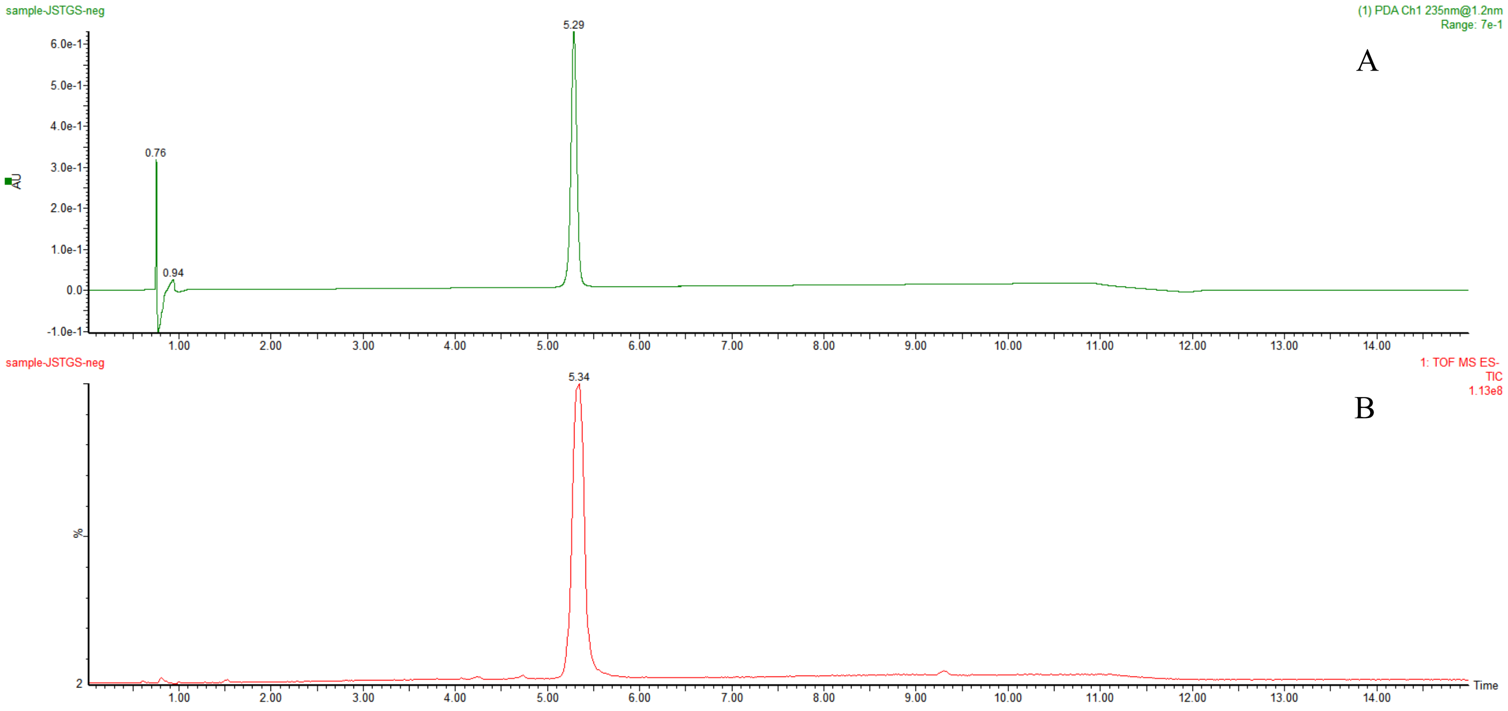

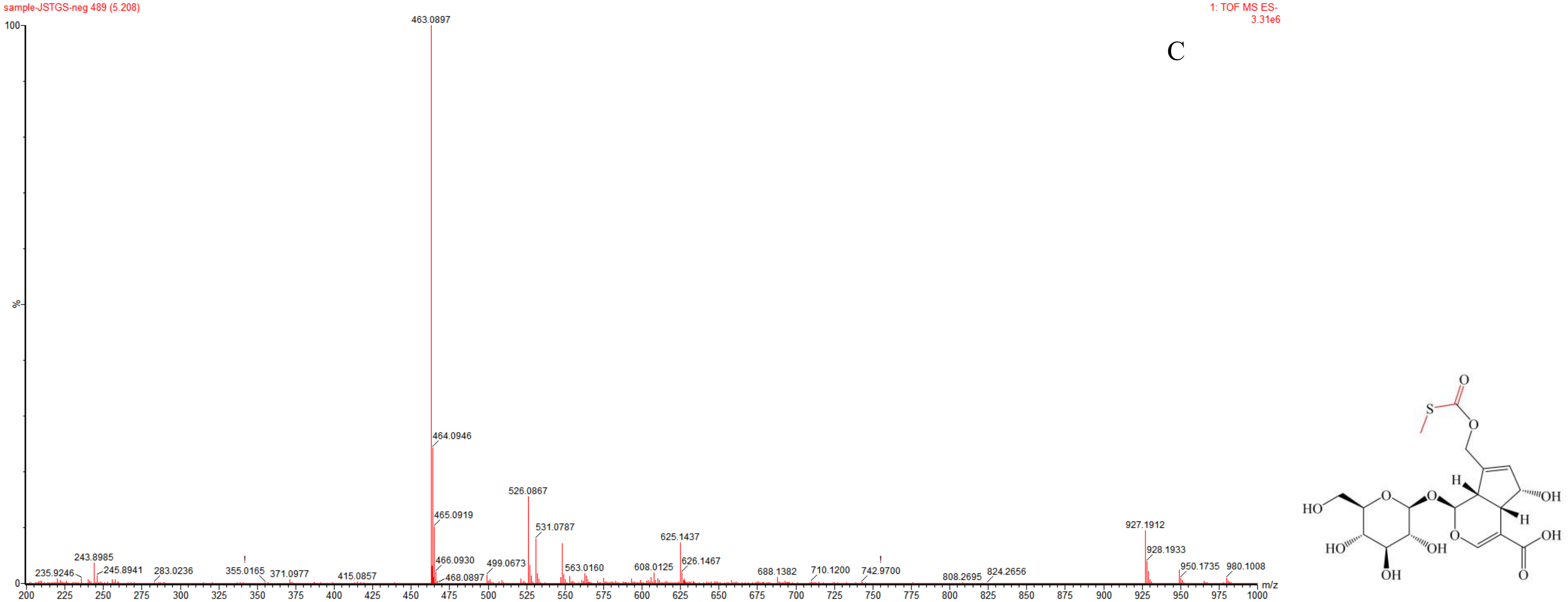

Figure S10 The UPLC-Q-Tof-MS Chromatograms of Paederosidic acid (JST-1) on Negative Ionization Mode. A: UV chromatogram at 235nm; B: Total ion chromatogram; C: Mass spectrum.

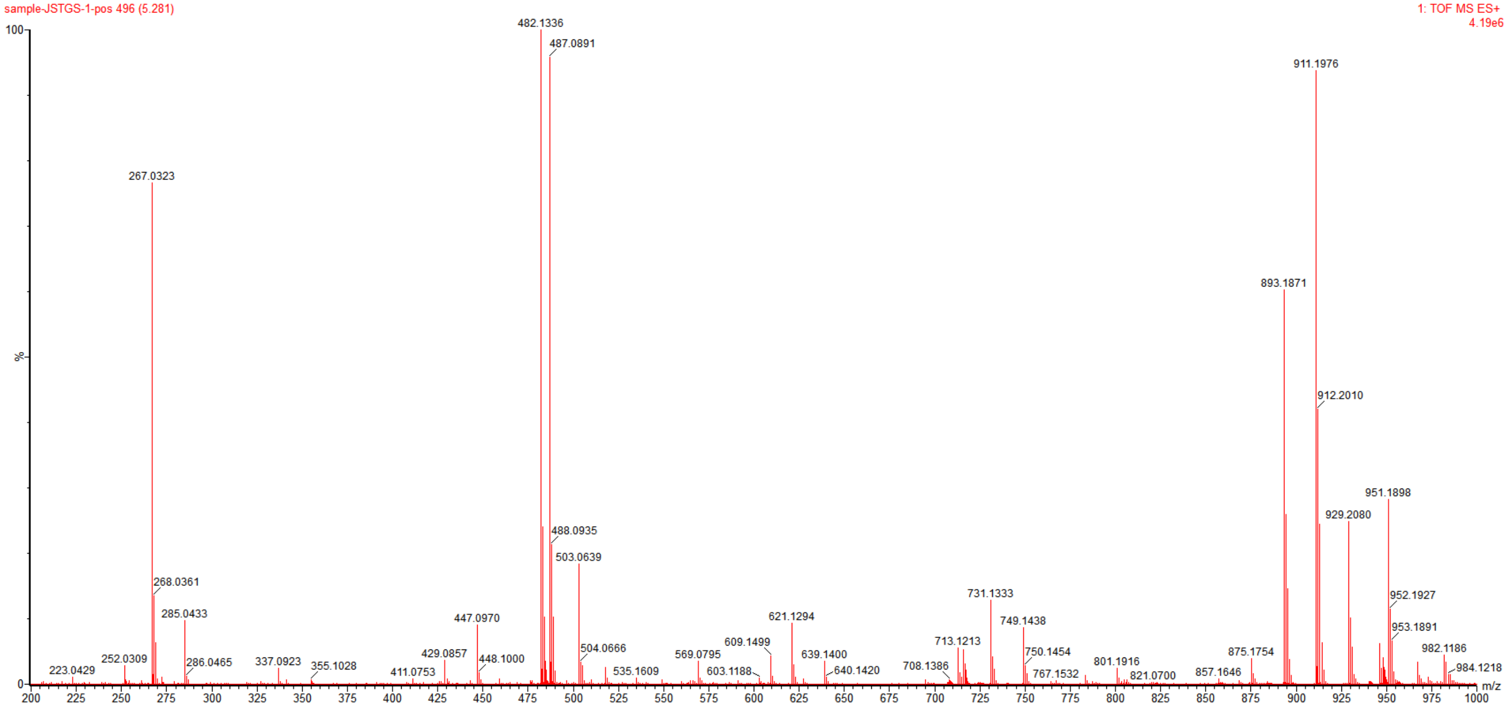

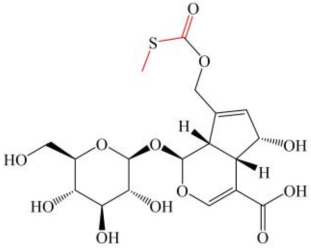

Figure S11 The mass spectrum of Paederosidic acid (JST-1) on Positive Ionization Mode.

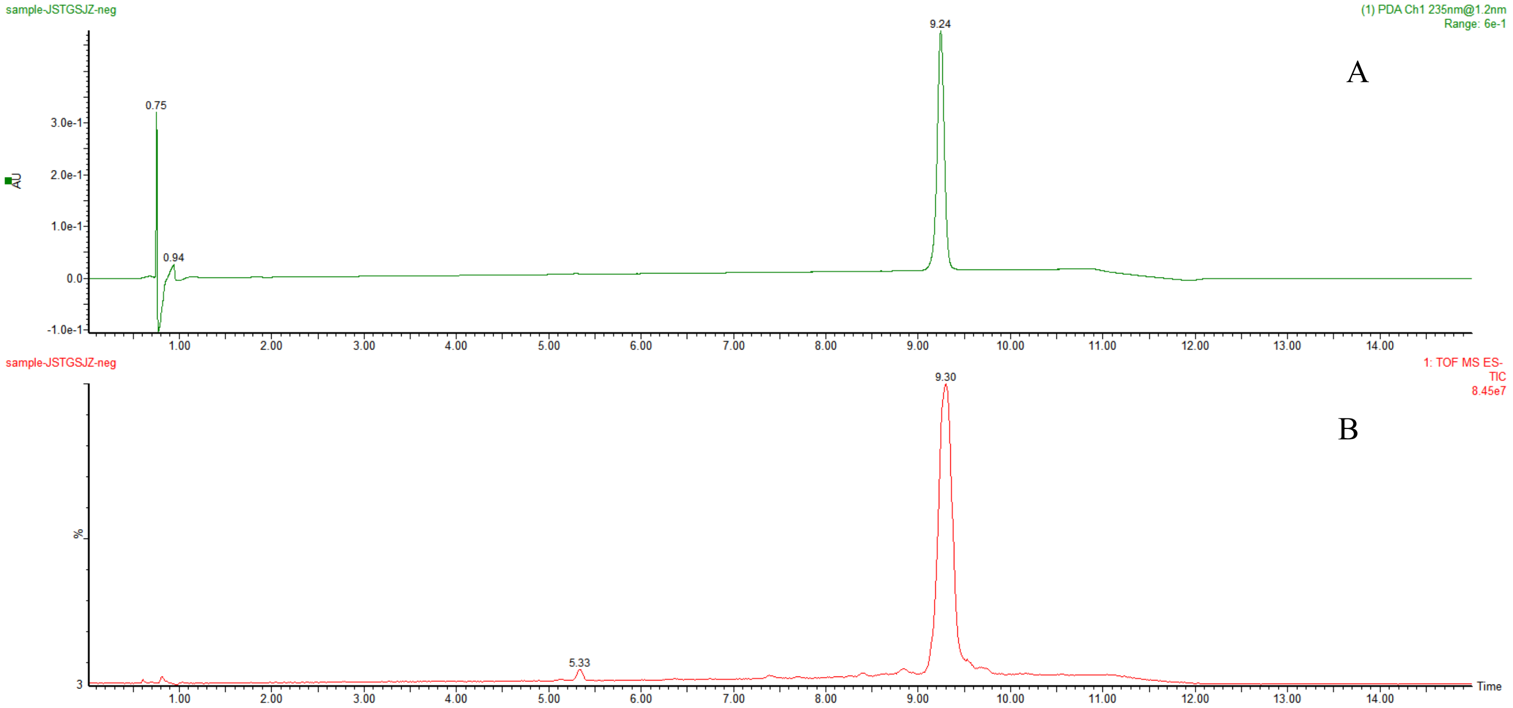

sample-JSTGSJZ-neg
(1) PDA Ch1 235nm@1.2nm
Range: 6e-1
A
AU
9.24
0.75
0.94
3.0e-1
2.0e-1
1.0e-1
0.0
-1.0e-1
1.00
2.00
3.00
4.00
5.00
6.00
7.00
8.00
9.00
10.00
11.00
12.00
13.00
14.00
sample-JSTGSJZ-neg
1: TOF MS ES-
TIC
8.45e7
B
%
9.30
5.33
3
Time

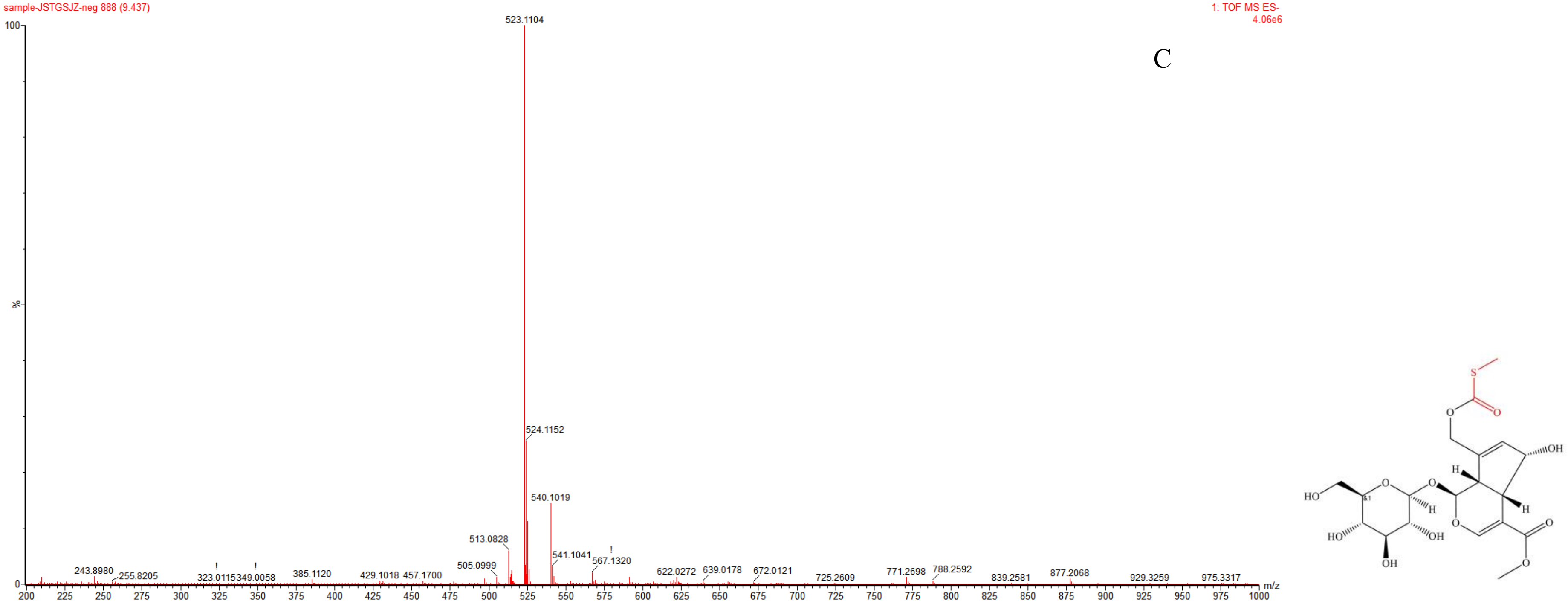

Figure S12 TThe UPLC-Q-Tof-MS Chromatograms of Paederosidic acid methyl ester (JST-2) on Negative Ionization Mode. A: UV chromatogram at 235nm; B: Total ion chromatogram; C: Mass spectrum.

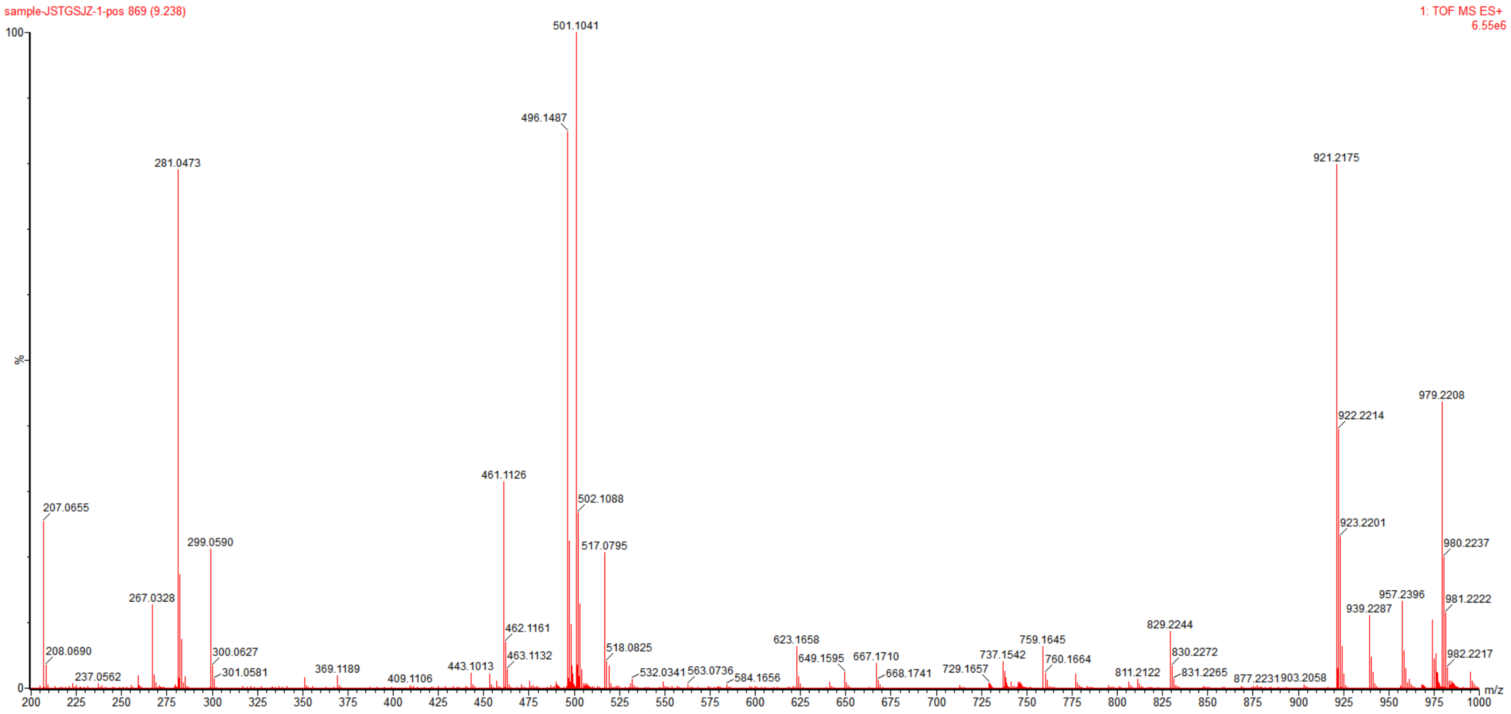

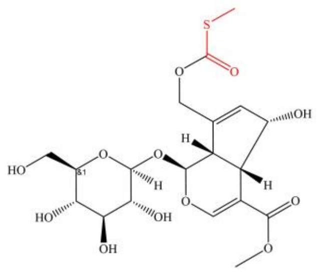

Figure S13 The mass spectrum of paederosidic acid methyl ester (JST-2) on Positive Ionization Mode.

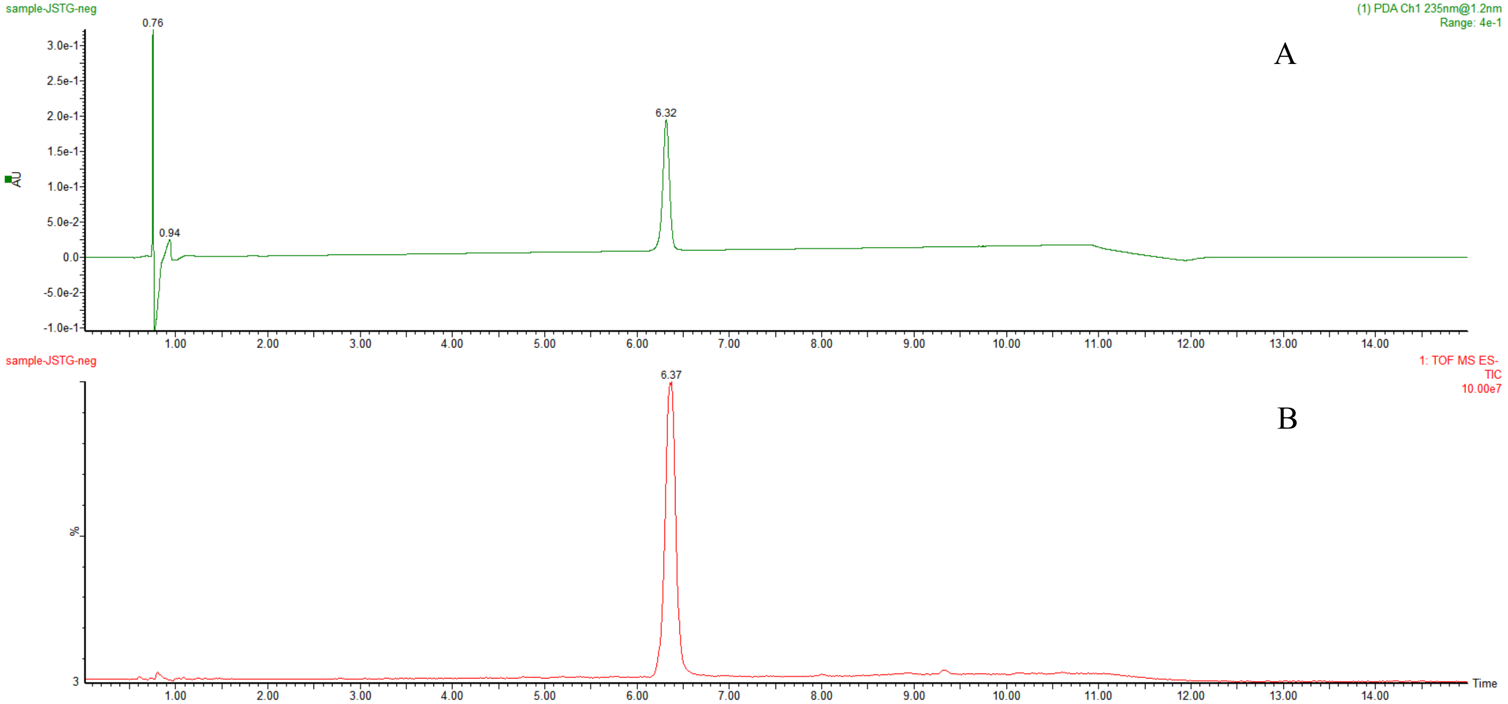

sample-JSTG-neg
(1) PDA Ch1 235nm@1.2nm
Range: 4e-1
A
0.76
0.94
6.32
AU
3.0e-1
2.5e-1
2.0e-1
1.5e-1
1.0e-1
5.0e-2
0.0
-5.0e-2
-1.0e-1
1.00
2.00
3.00
4.00
5.00
6.00
7.00
8.00
9.00
10.00
11.00
12.00
13.00
14.00
sample-JSTG-neg
1: TOF MS ES-
TIC
10.00e7
B
6.37
%
3
Time

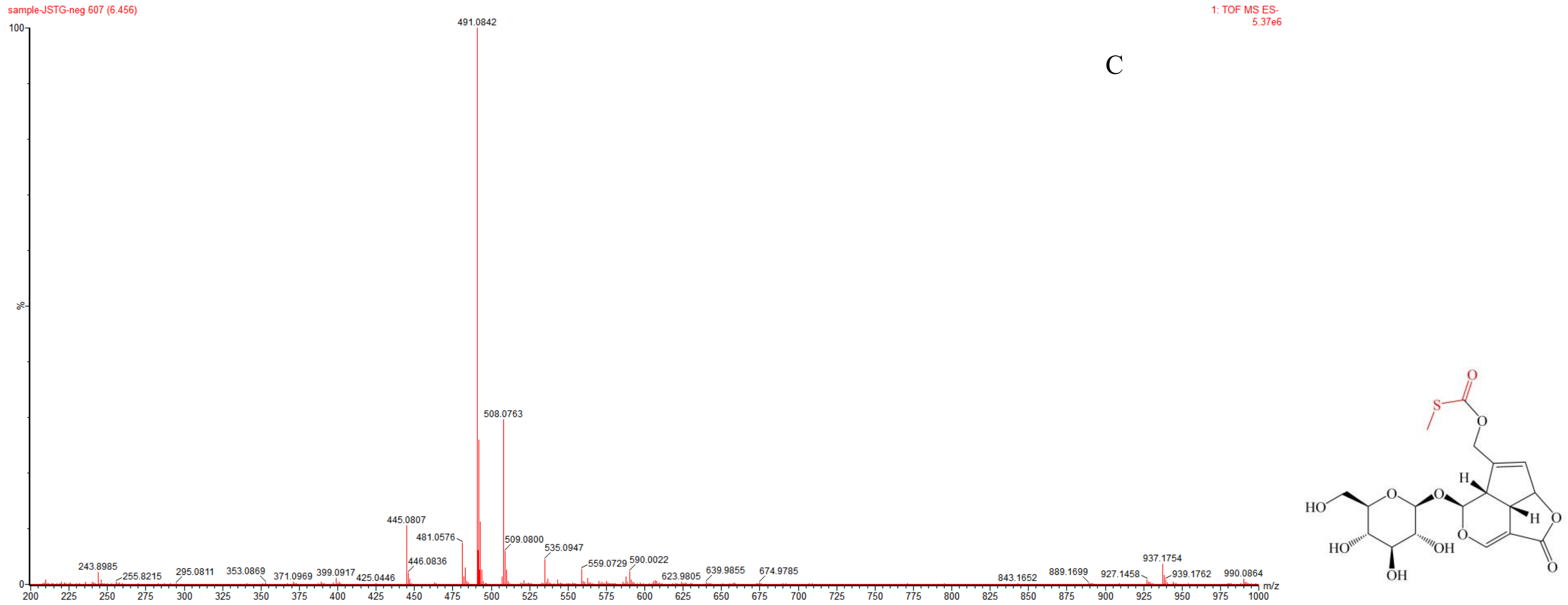

Figure S14 The UPLC-Q-Tof-MS Chromatograms of Paederoside (JST-3) on Negative Ionization Mode. A: UV chromatogram at 235nm; B: Total ion chromatogram; C: Mass spectrum.

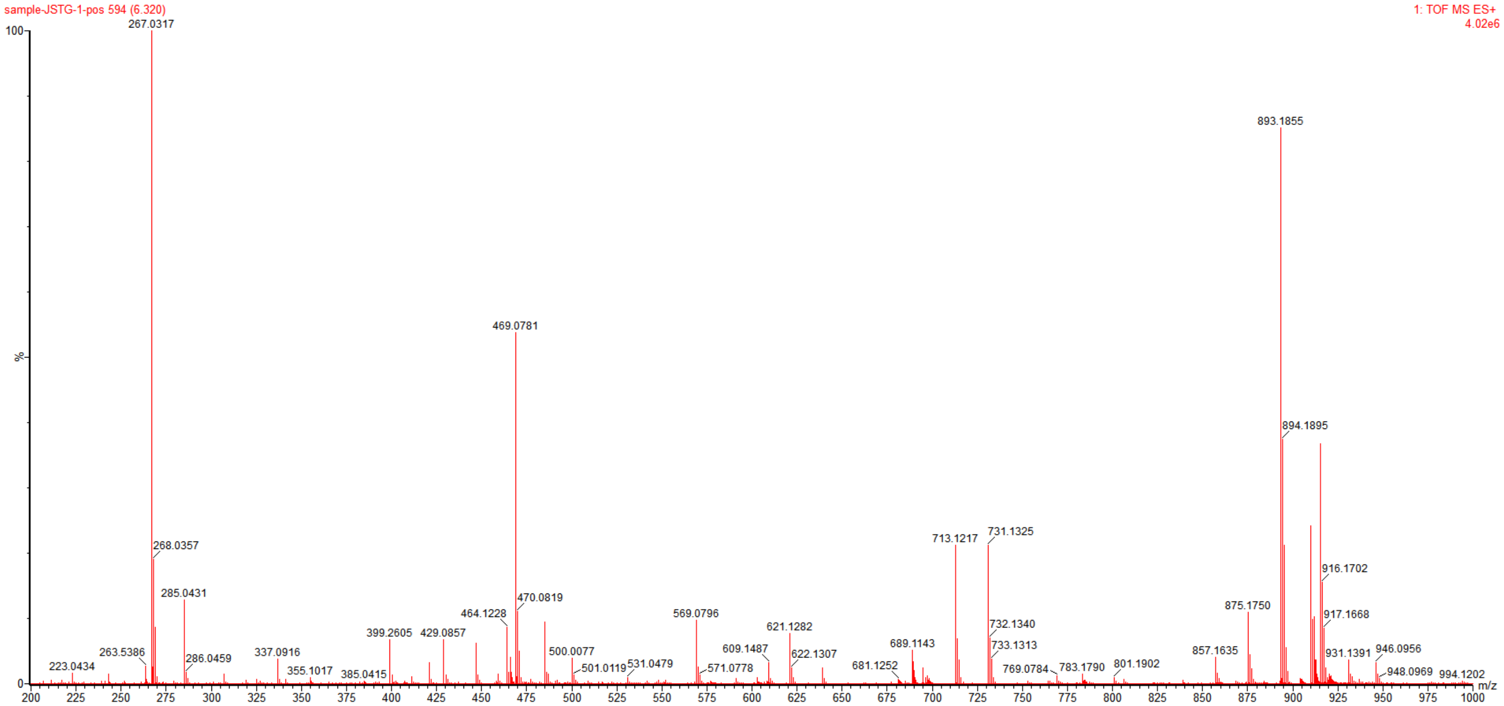

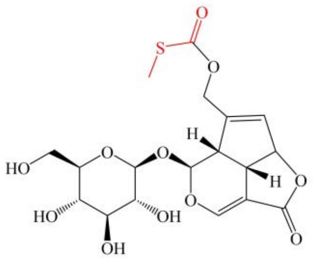

Figure S15 The mass spectrum of paederoside (JST-3) on Positive Ionization Mode.
